# Myoinositols Prevent Gestational Diabetes Mellitus and Related Complications: A Systematic Review and Meta-Analysis of Randomized Controlled Trials

**DOI:** 10.3390/nu15194224

**Published:** 2023-09-30

**Authors:** Dorina Greff, Szilárd Váncsa, Alex Váradi, Julia Szinte, Sunjune Park, Péter Hegyi, Péter Nyirády, Nándor Ács, Eszter Mária Horváth, Szabolcs Várbíró

**Affiliations:** 1 Centre for Translational Medicine, Semmelweis University, 1085 Budapest, Hungaryacs.nandor@med.semmelweis-univ.hu (N.Á.); 2 Department of Obstetrics and Gynecology, Semmelweis University, 1083 Budapest, Hungary; 3 Department of Physiology, Semmelweis University, Tűzoltó Str. 37-47, 1094 Budapest, Hungary; 4 Institute for Translational Medicine, Medical School, University of Pécs, 7621 Pécs, Hungary; 5 Institute of Pancreatic Diseases, Semmelweis University, 1085 Budapest, Hungary; 6 Department of Metagenomics, University of Debrecen, 4032 Debrecen, Hungary; 7 Department of Laboratory Medicine, Medical School, University of Pécs, 7621 Pécs, Hungary; 8 Department of Urology, Semmelweis University, 1083 Budapest, Hungary; 9 Workgroup for Science Management, Doctoral School, Semmelweis University, 1085 Budapest, Hungary; 10 Department of Obstetrics and Gynecology, University of Szeged, 6725 Szeged, Hungary

**Keywords:** GDM, inositol, preterm birth, neonatal, maternal health, gestational hypertension, preeclampsia

## Abstract

Although gestational diabetes mellitus (GDM) has several short- and long-term adverse effects on the mother and the offspring, no medicine is generally prescribed to prevent GDM. The present systematic review and meta-analysis aimed to investigate the effect of inositol supplementation in preventing GDM and related outcomes. Systematic search was performed in CENTRAL, MEDLINE, and Embase until 13 September 2023. Eligible randomized controlled trials (RCTs) compared the efficacy of inositols to placebo in pregnant women at high risk for GDM. Our primary outcome was the incidence of GDM, whereas secondary outcomes were oral glucose tolerance test (OGTT) and maternal and fetal complications. (PROSPERO registration number: CRD42021284939). Eight eligible RCTs were identified, including the data of 1795 patients. The incidence of GDM was halved by inositols compared to placebo (RR = 0.42, CI: 0.26–0.67). Fasting, 1-h, and 2-h OGTT glucose levels were significantly decreased by inositols. The stereoisomer myoinositol also reduced the risk of insulin need (RR = 0.29, CI: 0.13–0.68), preeclampsia or gestational hypertension (RR = 0.38, CI: 0.2–0.71), preterm birth (RR = 0.44, CI: 0.22–0.88), and neonatal hypoglycemia (RR = 0.12, CI: 0.03–0.55). Myoinositol decrease the incidence of GDM in pregnancies high-risk for GDM. Moreover, myoinositol supplementation reduces the risk of insulin need, preeclampsia or gestational hypertension, preterm birth, and neonatal hypoglycemia. Based on the present study 2–4 g myoinositol canbe suggested from the first trimester to prevent GDM and related outcomes.

## 1. Introduction

Gestational diabetes mellitus (GDM), defined as glucose intolerance newly recognized during pregnancy, is one of the most common pregnancy-related disorders [1]. While the prevalence of GDM varies based on population and diagnostic criteria, it is estimated to be around 14–16% globally [2,3,4].

GDM can lead to several maternal and offspring complications both in short and long-term, such as gestational hypertension, neonatal hypoglycemia, higher chance for obesity and type-2 diabetes mellitus (T2DM) in later life [5,6]. Moreover, women with GDM have higher risk of pancreatic cancer later in life [7]. Despite the serious complications, the current management of GDM focuses on the therapy of the already diagnosed GDM cases, and no generally accepted medical treatment is recommended to prevent GDM. The prevention of GDM would provide life-long advances for the mother and the offsprings, hence prevention of GDM should be a new focus in pregnancy care.

In the last few years, several studies investigated the beneficial effect of vitamin D, zinc, probiotics, dietary fibre, and lifestyle interventions in the prevention of GDM [4,8,9]. However, these attempts did not result in a breakthrough in prevention strategies. Inositol administration in early pregnancy is another novel preventive approach for GDM. Inositols are cyclic polyols. They can be found naturally in vegetables, fruits, nuts, and whole grains, but it can be synthesized in human body, too [10,11]. Inositols have nine stereoisomers, out of which myoinositol (MI) and D-chiro-inositol (DCI) are the most common forms utlilized. Inositols are insulin sensitizer, having an important role in insulin signal transduction pathways.

The beneficial effect of inositols were also examined in patients with polycystic ovary syndrome (PCOS) that is also commonly accompanied by insulin resistance. In insulin resistance, higher insulin levels leads to reduced synthesis and secretion of sex hormone binding globulin (SHBG) levels by the liver [12]. This results higher bioavailable testosterone levels worsening hyperandrogenic symptoms. Moreover hyperinsulinemia stimulates androgen overproduction in theca cells [13]. In these patients, they were shown to have beneficial effect on carbohydrate metabolism, menstrual cycle regularity, and hyperandrogenism [13]. During the course of healthy pregnancy, due to endocrine changes, insulin resistance develops and leads to hyperinsulinemia. GDM develops when β-cell function is insufficient to overcome the chronic insulin resistance associated with pregnancy [14]. Early administration of myoinositol may improve insulin resistance by encouraging the translocation of GLUT4 to the plasma membrane to increase glucose uptake [15]. Myoinositol serves as a structural basis of secondary messengers, such as inositol triphosphates (IP3) [16]. IP3 has a role in follicule stimulating hormone (FSH), thyroid stimulating hormone (TSH) and insulin signaling pathway [17]. Under insulin stimulation DCI is converted into MI by a unidirectional tissue-specific epimerase [18]. This reaction allows every tissue to develop a proper balance between DCI and myoinositol, ensuring normal metabolic functions [19]. As inositols play role in various steps of insulin signaling pathway, they can improve insulin resistance regardless of its origin. On the other hand, other present preventions are only effective in specific patients’ populations (e.g., vitamin D supplementation is only effective in vitamin D deficient patients).

At the time of PROSPERO registration, the last meta-analysis was 2 years old, and the quality of evidence was low. Since then, a few more RCTs have been published on this topic. In light of these, the present meta-analysis and systematic review aimed to investigate the effect of different inositols in preventing GDM and its complications.

## 2. Material and Methods

Our systematic review and meta-analysis is reported based on the recommendation of the PRISMA 2020 guideline [20] (see Table S1 for checklist), while we followed the Cochrane Handbook [21]. Furthermore, the study protocol was registered on PROSPERO (registration number CRD42021284939), and we fully adhere to it.

### 2.1. Eligibility Criteria

We used the PICO framework to formulate our research questions. Eligible randomized controlled trials (RCT) compared inositol supplementation (I) to placebo (C) in pregnant women (P) to prevent GDM or other GDM-related outcomes (O). There were no prespecified exclusion criteria regarding the pregnant population and the used inositol intervention. Eligible inositol supplementations were: myoinositol and/or D-chiro inositol–alone or in combination with other dietary supplements. Comparators were no intervention or placebo (e.g., dietary supplements, etc.).

The primary outcome was the diagnosis of GDM based on the diagnostic OGTT (by the 28th gestational week). Due to the the changes and regional differences of OGTT algorithm and glucose concentration thresholds, no study was excluded due to the method of diagnosis.

Secondary outcomes were the results of OGTT (fasting, 1- and 2-h post-load plasma glucose concentration), need for insulin treatment, preeclampsia, gestational hypertension, C-section, preterm birth, gestational age at birth, macrosomia, large for gestational age (LGA), birth weight, intrauterine growth restriction (IUGR), shoulder dystocia, neonatal hypoglycemia, diabetic fetopathy, and neonatal intensive care unit admission. Gestational hypertension and preeclampsia or pregnancy- induced hypertension data were handled as one outcome: pregnancy-related hypertensive disorders.

We only included RCTs in the present study, excluding reviews, non-randomized interventional studies, cohorts, case-controls, case reports, and case series.

### 2.2. Information Sources and Search Strategy

We conducted the systematic search on 5 November 2021, in MEDLINE (via PubMed), Embase, and Cochrane Central Register of Controlled Trials (CENTRAL). The systematic search was refreshed on 15 December 2022 and 13 September 2023 in the same search engines.

During the systematic search, the following search key was applied in all databases: (“gestational diabetes” OR GDM OR “gestational diabetic” OR “gestational diabetes mellitus” OR pregnancy OR LGA OR macrosomia OR “large for gestational age”) AND (inositol* OR myoinositol OR chiroinositol OR DCI). No study type, date, or language filters were applied during the search.

### 2.3. Selection Process

The selection was performed by two independent review authors using the Endnote X9 (Clarivate Analytics, Philadelphia, PA, USA) reference manager program. After the duplication removal process, articles were selected based on title and abstract, then based on full text. A third review author resolved disagreements during the selection process. We used Cohen’s kappa to measure inter-rater reliability [22]. Cohen’s kappa was 0.87 at selection by title and abstract. At the phase of full text selection Cohen’s kappa was 0.97. All disagreements were solved by a third reviewer.

### 2.4. Data Collection Process and Data Items

Data from the eligible RCT were collected into a standardized data collection sheet by two authors independently. All disagreements were solved by a third reviewer. The following data were extracted from the eligible articles: title, first author, year of publication, countries, study design, main study findings, patient demographics, interventions, outcomes: maternal complications (OGTT: fasting, 1 and 2 h post-load plasma glucose concentration, insulin treatment, preeclampsia, gestational hypertension, mode of delivery: C-Section rate) and fetal complications (preterm birth, gestational age at birth, macrosomia, LGA, birth weight, IUGR, shoulder dystocia, neonatal hypoglycemia, diabetic foetopathy, neonatal intensive care unit admission).

For dichotomous data, we extracted the events for the outcomes and total numbers of patients in the intervention and control groups. In addition, mean and standard deviation (SD) values were collected in both groups for continuous variables.

### 2.5. Study Risk of Bias Assessment

Two review authors assessed the quality of the included studies using the Cochrane risk-of-bias tool for randomized trials (RoB 2) [23]. A third review author resolved disagreements. Bias was evaluated in five main domains: randomization process, deviations from intended interventions, missing outcome data, measurement of the outcome, and selection of the reported results.

### 2.6. Assessing the Level of Evidence

The quality of evidence was evaluated according to the recommendation of the “Grades of Recommendation, Assessment, Development, and Evaluation (GRADE)” workgroup [24].

### 2.7. Synthesis Methods

In the case of sufficiently homogenous studies based on the PICO, we performed both qualitative and quantitative data synthesis. The minimum number of studies to perform a meta-analysis was three.

All statistical calculations were done using the R programming language (R Core Team, 2022, Vienna, Austria, R v4.2.1) using the meta v6.0-0, metafor v3.8-1 and dmetar v0.0.9000 packages [25,26,27]. The quantitative results were presented by calculating mean differences (MD) with 95% confidence intervals (CIs) for continuous variables. In the case of dichotomous outcomes, we calculated risk ratios (RRs) with 95% CIs. All the analyses were carried out using the random-effects models and displayed on forest plots. To pool binary outcome data such as like Preterm birth, we applied the Mantel-Haenszel method with the Paule-Mandel method to estimate the between-study variance [28,29,30]. However, in case of continous outcomes, like birthweight, we used Restricted maximum likelihood methods (REML) to estimate the between-study variance and inverse variance for weighting [31]. A *p* < 0.05 was considered statistically significant. Where applicable prediction intervals of the pooled estimates were also been reported [32].

Statistical heterogeneity was tested by the *I*² statistics and the Cochrane Q test; *p* < 0.1 indicated significant heterogeneity. Furthermore, publication bias could not be assessed due to the low number of studies.

We performed subgroup analysis based on inositol stereoisomers.

## 3. Results

### 3.1. General Characteristics of the Studies

**Figure 1:**
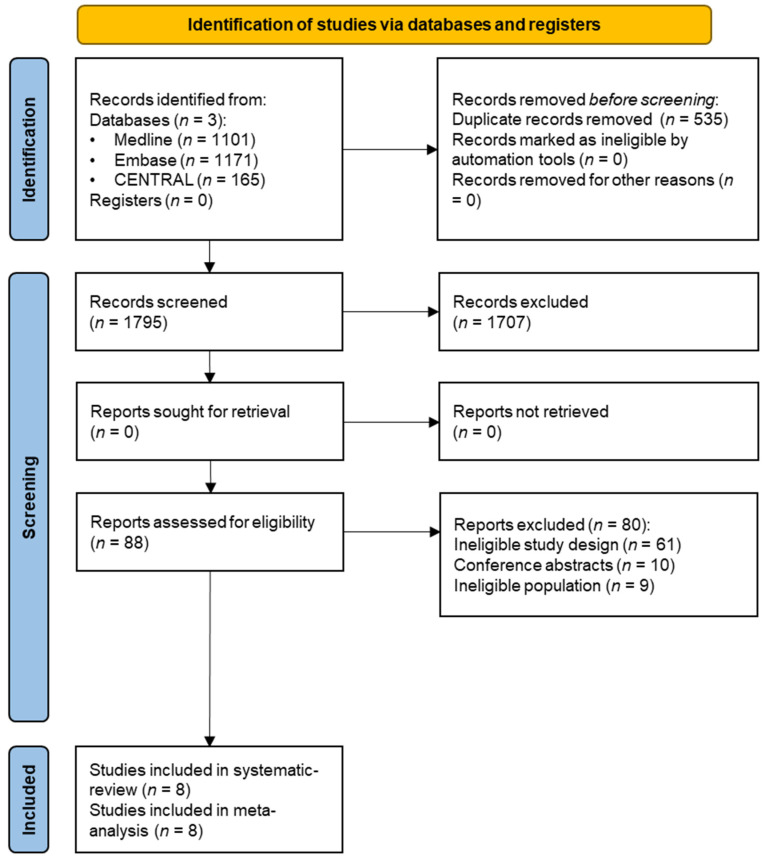
PRISMA 2020 flowchart representing the study selection process.

After the duplication removal, we screened 1795 references by title and abstract. Secondly, 88 articles were screened by full text. At the end of the selection process, eight RCTs with 1361 pregnant women were eligible to be included in this meta-analysis (Figure 1) [33,34,35,36,37,38,39,40].

The baseline characteristics of the enrolled analyses are detailed in Table 1. Altogether, 515 pregnant women received myoinositol supplementation, 32 patients received DCI supplementation, 154 patients received the combination of myoinositol and DCI. Placebo was administered in case of 660 pregnant women. One RCT investigated myoinositol, DCI and inositol combination separately [33]. Six RCTs compared myoinositol administration to placebo and one RCT investigated the beneficial effect of the combination of myoinositol, and DCI compared to placebo [37].

The diagnosis of GDM was based on the International Association of Diabetes and Pregnancy Study Groups (IADPSG) recommendations in every RCTs and all the studies started the inositol supplementation at the 12th–13th gestational week. Recommendations of IADPSG state that GDM is diagnosed when one of the fasting, 1 h and 2 h post-load glucose level after consuming 75 g glucose, was more than the expected threshold of 92, 180 and 153 mg/dl respectively between 24th and 28th gestational week [3].

All eligible studies included patients with high risk for GDM. Four of them investigated overweight [36,39,40] and obese patients [34]. Matarelli [38] and Celenatano [33] examined pregnant women with elevated first-trimester blood glucose, meanwhile, two other RCTs studied pregnant women with a family history of type-1 or type-2 diabetes [35,37]. Inclusion and exclusion criteria are summarized in Table S2.

### 3.2. Synthesis of the Results

#### 3.2.1. Inositols Decrease the Occurrence of GDM

**Figure 2:**
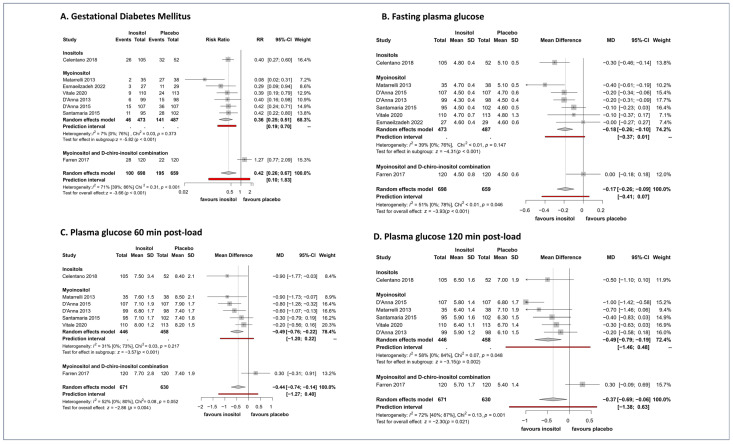
(**A**) Forest plots representing the risk of developing GDM; (**B**) Forest plots representing the mean differences of fasting glucose; (**C**) Forest plots representing the mean differences of 1 h-OGTT; (**D**) Forest plots representing the mean differences of 2 h-OGTT.3.3. Maternal health outcomes. (Pooled results are presented by the diamond in the bottom. Prediction interval is presented by red line) [33,34,35,36,37,38,39,40].

Overall, 1357 pregnant women were included in the analysis of GDM occurrence. Inositol administration starting from the 12th-13th gestational week significantly reduced the risk of GDM (RR = 0.42, CI: 0.26–0.67) compared to placebo (Figure 2A). Seven RCTs investigated myoinositol supplementation, and all the studies showed that myoinositol could significantly reduce the risk of GDM (RR = 0.3, CI: 0.18–0.48). We found only one article regarding DCI administration, suggesting it has a beneficial effect on GDM prevention (RR = 0.56, CI: 0.33–0.94). Based on two studies, the combination of myoinositol and DCI showed no benefit in preventing GDM compared to placebo (RR = 0.89, CI: 0.44–1.79) (Figure S1).

#### 3.2.2. Inositol Reduces Fasting, 60′, and 120′ Glucose Levels during OGTT

Inositol supplementation significantly reduced the fasting glucose level at the 24th–28th gestational week (MD = −0.17 mmol/L, CI: −0.26; −0.09) (Figure 2B). In the 60′ and 120′ post-load plasma glucose levels, a significant difference was observed in favor of inositols compared to placebo. It decreased OGTT 60′ glucose level on the average by MD = −0.44 mmol/L (CI: −0.74; −0.14) (Figure 2C) and it decreased OGTT 120′ glucose level by MD = −0.37 mmol/L (CI: −0.69; −0.06) (Figure 2D).

Based on the subgroup analysis, myoinositol significantly reduced all glucose levels during OGTT. Fasting glucose concentration was decreased on the average by MD = −0.21 mmol/L (CI: −0.30; −0.11), 1 h post-load glucose level was decreased on the average by MD = −0.53 mmol/L (CI: −0.79; −0.27), and 2 h post-load glucose concentration was decreased on the average by MD = −0.50 mmol/L (CI: −0.77; −0.23) by myoinositol (Figure S2–S4).

**Figure 3:**
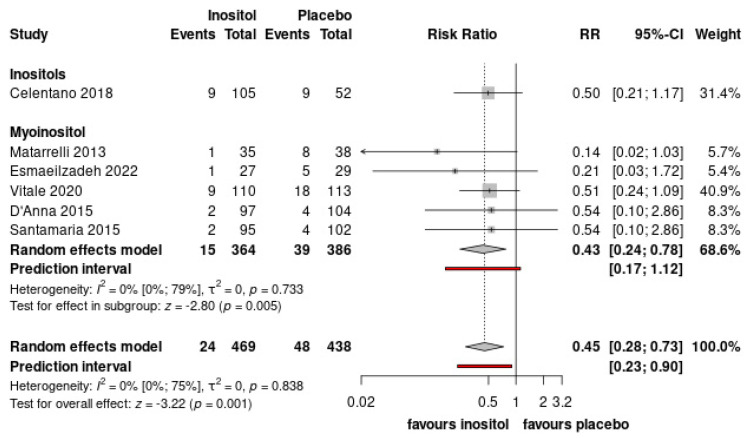
Forest plots representing the risk of insulin need. (Pooled results are presented by the diamond in the bottom. Prediction interval is presented by red line) [33,34,36,38,39,40].

**Figure 4:**
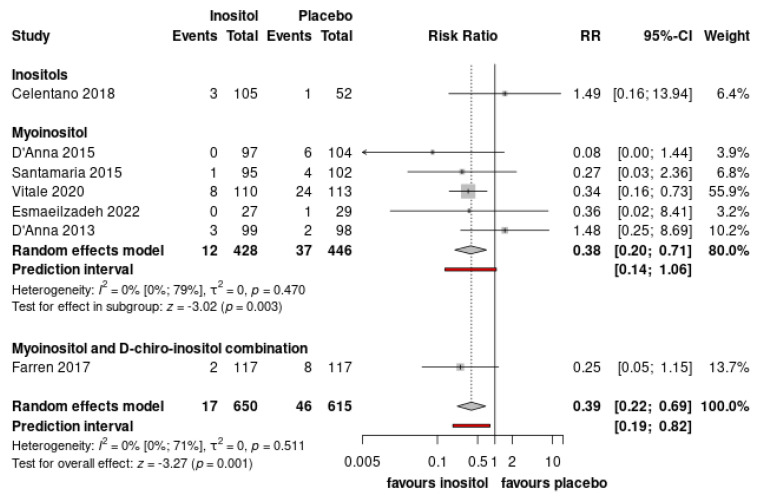
Forest plots representing the risk of pregnancy-induced hypertensive disorders. (Pooled results are presented by the diamond in the bottom. Prediction interval is presented by red line) [33,34,35,36,37,39,40].

The number of patients in need for insulin treatment was significantly lower in the inositol-treated group compared to the placebo (RR = 0.45, CI: 0.28–0.73) (Figure 3, Figure S5). Preeclampsia or pregnancy-induced hypertensive disorders was also significantly lower in the inositol-treated group compared to the non-treated group (RR = 0.39 CI: 0.22–0.69) (Figure 4, Figure S6). Of the eight included RCTs, all studies investigated the incidence of side effects. Only Esmaeilzadeh et al. reported side effect (headache) in one patient [36].

### 3.3. Delivery Outcomes

**Figure 5:**
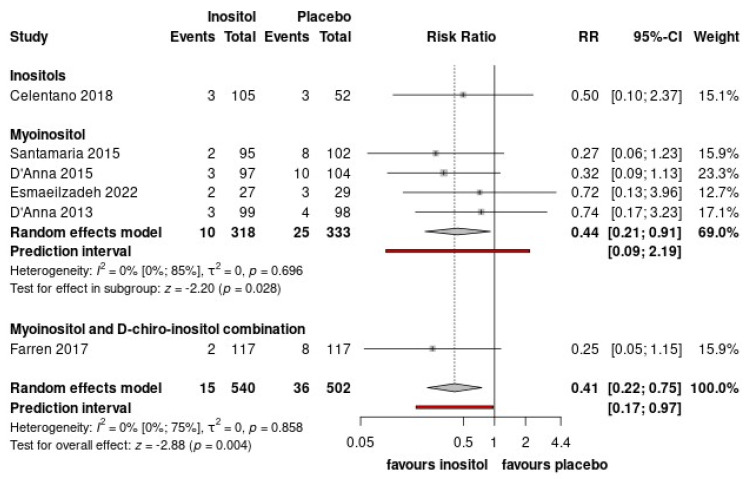
Forest plots representing the risk of preterm birth. (Pooled results are presented by the diamond in the bottom. Prediction interval is presented by red line) [33,34,35,36,37,39].

Based on the present analysis, inositol supplementation significantly reduces the risk of preterm birth (RR = 0.41, CI: 0.22–0.75) (Figure 5, Figure S7). However, no significant difference was observed regarding gestational age at birth (MD = 0.52, CI: −0.03; 1.08). The combination of myoinositol and DCI might favor gestational age at birth (MD = 0.36, CI: 0.00–0.71) (Figure S8). However, this is based only on the results of one study. No significant difference was found in the incidence of C-section (RR = 0.90, CI: 0.78–1.03) and the risk of shoulder dystocia (RR = 0.59, CI: 0.12–2.82) between in the intervention and control group (Figures S9 and S10).

### 3.4. Fetal-Neonatal Health Outcomes

**Figure 6:**
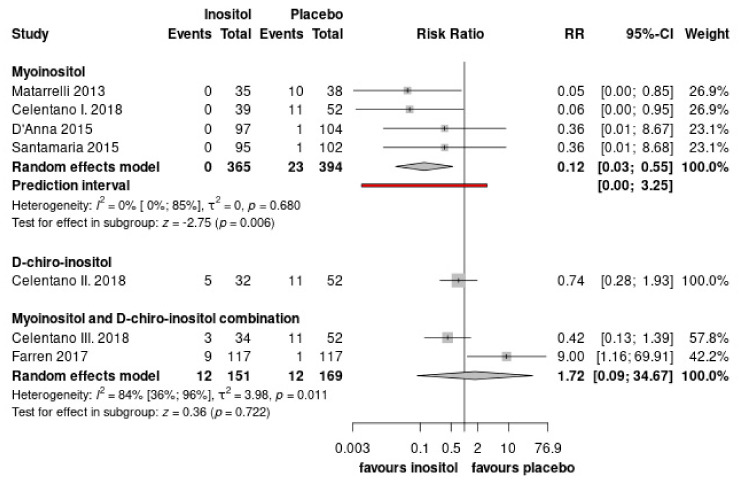
Shows the overall effect on neonatal hypoglycemia. (Pooled results are presented by the diamond in the bottom) [33,34,37,38,39].

Six RCTs investigated birthweight, and five RCTs evaluated macrosomia, suggesting that inositol supplementation does not affect them (Figures S11 and S12). Regarding neonatal hypoglycemia, we found that myoinositol has a beneficial effect on it and significantly reduces the risk of hypoglycemia (RR = 0.12, CI: 0.03–0.55). (Figure 6, , Figure S13) However, neonatal intensive care unit (NICU) admission was reported in five studies showing inositol did not affect this outcome (Figure S14). Lastly, insufficient data were found regarding IUGR, and diabetic fetopathy. Only Celentano et al. reported on LGA, suggesting that myoinositol may have beneficial influence on that outcome [33].

Overall for GDM, 2 h-OGTT, gestational age at birth, neonatal hypoglycemia outcomes the heterogeneity was high, while for insulin therapy, C-section rate, neonatal intensive care unit admission, preterm birth, shoulder dystocia and gestational hypertension outcomes, it was low. The source of heterogeneity is the different inositol stereoisomers used in different dosages (2–4 g MI or 500 mg DCI or 27.6 mg DCI and 1100 mg MI in combination) and the variable BMI in the included population. The results of the risk of bias assessment are presented in Table S3. Moderate risk of bias resulted from the lack of blinding methods. Only Matarelli’s and Esmaeilzadeh’s RCTs are double-blinded [36,38]. The others were open-labeled studies.

The level of evidence varies from very low to moderate. The level of evidence was high in case of one outcome: preterm birth. The level of evidence is summarized in Table S4.

## 4. Discussion

A higher frequency of obesity and glucose metabolism disorder in adolescents and children has increased the probability of T2DM in fertile patients [41,42]. Therefore, the higher prevalence of T2DM in women of fertile age results in a higher prevalence of GDM [2]. The long-term adverse effects of GDM are a higher risk for T2DM and associated cardiometabolic risk for both mothers and offspring. According to Vounzoulaki et al., mothers who suffer from GDM have almost tenfold higher risk of T2DM, and Kramer et al. reported that these patients have two-fold higher risk of cardiovascular events [5,6,43]. Similar results were described for the offsprings, also [44]. During pre-pregnancy care to prevent GDM, the risk factors of the condition should be eliminated. Inositol supplementation is also beneficial in PCOS treatment, as one of the risk factors of GDM [45,46].

Despite of the serious life-long consequences and the several studies on interventions to prevent GDM no generally accepted medical treatment is recommended to prevent GDM in the latest guideline. Based on the present results, inositol supplementation can reduce the prevalence of gestational diabetes. Moreover, inositol can reduce the need for insulin treatment and the risk of pregnancy-induced hypertensive disorders, preterm birth and neonatal hypoglycemia. Inositols significantly decreased the fasting, 1-h, and 2-h OGTT glucose levels. We found no significant effect regarding other examined parameters, however; most of them had low event rates and were reported in low number of patients (e.g., macrosomia, NICU admission, shoulder dystocia).

*Inositol supplementation administered from the first trimester can prevent the development of GDM by decreasing fasting, 1-h, and 2-h OGTT glucose levels.*

Upon our data, preventive inositol supplementation started before the 13th week of pregnancy, halved the risk of GDM during the pregnancy. Since the diagnosis of GDM is based on OGTT, it is not surprising that fasting, 1-h, and 2 h-OGTT glucose levels showed a similar decrease by the end of the 28th week.

Previous meta-analyses and systematic reviews had similar conclusions to the present work [16,47,48,49,50]. Both Wei et al., Guo et al., Chan et al. and the Cochrane review presented the significant beneficial effect of myoinositol supplementation in preventing GDM [16,47,48,49]. The preventive effect of both, myoinositol and DCI were investigated by Vitagliano et al. [50] Wei et al. pooled all included studies in one group of “4 g MI group”, and although in one study only 2 g MI was administered (Vitale et al.), they still concluded the positive effect of inositol supplementation [16,40].

Based on Farren et al.’s RCT, the combination of myoinositol and D-chiro-inositol seemed to have no effect in the prevention. It is possible that the combination of the two inositol stereoisomers is not effective, or the used dose of inositol, especially MI was too low (1100 mg MI + 27.6 mg DCI) [37]. The five RCTs that administered 4 g of myoinositol, found the supplementation useful in primary prevention [33,34,35,37,39]. Vitale et al. and Esmaeilzadeh et al. administered 2 g of myoinositol which was also beneficial [36,40]. According to the available data, the proper dose of myoinositol is between 2 and 4 g per day in the prevention of gestational diabetes—so we conclude that over 2 g, myoinositol can halve the risk of GDM. However, in high-risk patients with diabetes in family history, 1100 mg MI + 27.6 mg DCI is an ineffective combination for preventing GDM.

Prevention of GDM is one of the biggest challenge of prenatal care. Several promising strategies emerged in the recent years, mainly focusing on life-style intervention (diet and physical exercise) and dietary supplements besides inositol, like vitamin D and magnesium, and probiotics. According to a recent network meta-analysis; physical exercise (OR: 0.64 (0.46–0.88)) and probiotic intake (OR: 0.57 (0.34–0.96)) was able to reduce the risk of GDM. Same analysis failed to show the protective effect of inositols, most probably due to the inclusion of only four studies including Farren et al. with 1100 mg MI + 27.6 mg DCI intake [51]. GDM is a multietological disorder. If one potential risk factor is cured, it doesn’t necessary influence other etiological factors contributing to the development of GDM. For example, magnesium supplementation was found effective in magnesium-insufficient patient. Similarly, patients suffering from vitamin D supplementation might benefit the most from vitamin D supplementation [4]. To overcome this problem, various combined treatments were also tested for the prevention of GDM. D’ell Edera et al. investigated the combination of myoinositol, DCI, zinc, methylsulfonylmethan, and methyltetrahydrofolic acid. They reported that the abovementioned combination might prevent the onset of GDM [52]. In contrary to these, Godfrey et al. reported that in a general population myoinositol 4 g/day, vitamin D 10 μg/day, riboflavin 1.8 mg/day, vitamin B6 2.6 mg/day, vitamin B12 5.2 μg/day, zinc 10 mg/day, and probiotics (Lactobacillus rhamnosus NCC 4007 and Bifidobacterium animalis species lactis NCC 2818,folic acid 400 μg/day, iron 12 mg/day, calcium 150 mg/day, iodine 150 μg/day, and β-carotene 720 μg/day vs. folic acid, iron, calcium, iodine, and β-carotene didn’t reduce the incidence of GDM if started prior conception [53]. These results are surprising as each of the additional supplements were shown to be effective separately before. One reason of the results could be the high versatility of the study population in GDM risk and ethnicity compared to previous studies mostly including high-risk Caucasian cohorts. Another possibility is the interaction of the supplements. For example, folic acid supplementation was linked to increased GDM risk especially in case of vitamin B12 insufficiency. And they also observed relatively high prevalence of GDM (24.8% and 22.6%), despite the general study population, and the fact that patients with known type2 diabetes or taking metformin were excluded from the study [54].

*Inositol reduce the need for insulin treatment and the risk of pregnancy-induced hypertensive disorders (preeclampsia or gestational hypertension)*

Based on our data, if inositol supplementation was started before the 13th week of pregnancy, it halved the risk of need for insulin. Individual RCTs with low number of patients in need of insulin treatment [33,34,36,38,39,40] failed to show this effect. The meta-analyis by Wei et al. confirms our results regarding insulin treatment. However, Wei et al. showed no difference between inositol treated and placebo-treated groups regarding pregnancy-induced hypertensive disorders [16].

*Inositol supplementation lowers the risk of preterm birth and neonatal hypoglycemia.*

Vitagliano et al. in their meta-analysis concluded a significant beneficial effect of inositol in reducing preterm birth but no effect on neonatal hypoglycemia [50]. Wei et al. draw the same conclusion to our analysis that inositol could reduce the risk of preterm birth and neonatal hypoglycemia, too [16]. Although Godfrey et al. in their RCT failed to show the preventive effect of inositol administration in combination with other supplements on GDM prevalence, they still concluded that the combined treatment reduced the risk of preterm birth [53]. Interestingly, if GDM patients are treated with 4 g of myoinositol, the risk of preterm delivery and the need for insulin is reduced. Therefore, inositol supplementation is useful not only for preventing GDM but also for preventing the complications of the manifest disease [55,56].

### 4.1. Strengths and Limitations

Our study has several strengths and limitations. First, as a strength, the study protocol was strictly followed, which was registered in advance in PROSPERO. A rigorous methodology was applied. Not just different doses of myoinositol but also DCI, and the combination of myoinositol and DCI was investigated on several outcomes for the mother and fetus. Regarding the several different diagnostic criteria of GDM worldwide, the strength of the present work is that in all included RCTs the same criteria was used to diagnose GDM.

The study’s limitations are the small number of included studies and patients. As a result, some of our results have high heterogeneity. Mostly the source of heterogeneity is the different inositol stereoisomers used in different dosages. To resolve this problem subgroup analysis was reported. Additional heterogeneity comes from variable BMI. The lack of blinding methods is another limitation of the analyzed studies.

### 4.2. Implication for Practice and Research

The benefit of implementing scientific results into daily practice has been previously proved [57,58]. Based on our results, myoinositol 2–4 g/day supplementation should be used in high-risk pregnancies to reduce the risk of GDM and its complications. Based on our and a previous meta-analysis inositols are safe during pregnancy [16]. Its preventive effect may recommends its use in low-risk pregnancies [59].

However, further prospective data collection is needed to assess the effectiveness of inositol administration in pregnancies low-risk for GDM more accurately. The most effective dosages of inositol should be also investigated in the future in double-blinded studies. An international registry about prepregnancy inositol administration of high-risk patients might give additional insight regarding this topic. Insulin needs should be interpreted with the exact amount of insulin needed instead of a dichotomous format.

## 5. Conclusions

Inositol, especially myoinositol, halves the risk of GDM in high-risk pregnancies. In addition, inositols showed beneficial effects on several GDM-related outcomes. Myoinositol reduces the need of insulin treatment and the risk of preeclampsia or gestational hypertension, preterm birth, and neonatal hypogylcemia. Based on our meta-analyis in high-risk pregnancies daily 2–4 g myoinositol supplementation from the 12th–13th gestational week is suggested to prevent GDM and related outcomes.

## Figures and Tables

**Table 1:** Basic characteristics of included studies.

Author (Year)	Country	Number of Patients (I/C)	Age (Year) (I/C) ‡	BMI (kg/m^2^) (I/C) ‡	Risk Factors/Inclusion Criteria	Intervention	Outcomes	Baseline Fasting Glucose (mg/dL) ‡
Celentano, 2018 [33]	Italy	105/52	33.8/33.9	23.8/24.4	elevated fasting glucose at first trimester blood exams.	4 g MI + 400 mcg FA; 500 mg DCI + 400 mcg FA; 1100 mg MI + 27.6 mg DCI	GDM, OGTT, insulin therapy, preeclampsia or pregnancy-induced hypertension, C-section, preterm birth, neonatal hypoglycemia, NICU admission	97.2/97.2
D’Anna, 2013 [35]	Italy	99/98	31/31.6	22.8/23.6	family history of type 2 DM	4 g MI + 40 mcg FA	GDM, gestational hypertension, C-section, shoulder dystocia, preterm delivery, gestational age at delivery, neonatal hypoglycemia.	-
D’Anna, 2015 [34]	Italy	97/104	30.9/31.7	33.8/ 33.8	prepregnancy BMI 30 or greater	4 g MI + 40 mcg FA	GDM, OGTT, insulin treatment, gestational hypertension, C-section, shoulder dystocia, preterm birth, gestational age at delivery, macrosomia, birth weight, neonatal hypoglycemia, NICU admission	83.1/82.3
Esmaeilzadeh, 2022 [36]	Iran	27/29	27.8/29.3	27.3/26.9	overweight patients (prepregnancy BMI above 25 and under 30), age 18–40	2 g MI + 200 mcg FA	GDM, fasting blood sugar, fasting blood insulin, insulin treatment, preeclampsia or pregnancy-induced hypertension, shoulder dystocia, C-section, preterm delivery, NICU admission	84/85.2
Farren, 2017 [37]	Ireland	120/120	31.1/31.5	26/26.2	patients with a family history in a first-degree relative of diabetes, either type 1 or type 2.	1100 mg MI + 27.6 mg DCI + 400 mcg FA	GDM, OGTT, preeclampsia or pregnancy-induced hypertension, C-section, shoulder dystocia, preterm delivery, gestatational age at delivery, macrosomia, birth weight, hypoglycemia, NICU admission	-
Matarelli, 2013 [38]	Italy	35/38	33/33.8	23.5/24.7	elevated fasting glucose and BMI under 35	4 g MI + 400 mcg FA	GDM, OGTT, insulin therapy, gestational age at delivery, birth weight, neonatal hypoglycemia	97.2/97.2
Santamaria, 2015 [39]	Italy	95/102	32.1/32.7	26.9/27.1	overweight patients (prepregnancy BMI above 25 and under 30)	4 g MI + 400 mcg FA	GDM, OGTT, insulin treatment, gestational hypertension, shoulder dystocia, C-section, preterm delivery gestational age at delivery, macrosomia, neonatal hypoglycemia, NICU admission	81.08/78.63
Vitale, 2020 [40]	Italy	110/113	27.18/27.95	27/26.68	overweight patients (prepregnancy BMI above 25 and under 30)	4 g MI + 400 mcg FA	GDM, OGTT, gestational hypertension	82.2/83.1

‡ Parameters represented as mean. I/C—intervention and control group. BMI: body mass index; DCI: d-chiro-inositol; FA: folic acid; GDM: gestational diabetes; OGTT: oral glucose tolerance test; MI: myoinositol; NICU: neonatal intensive care unit.

## Data Availability

The data presented in this study are available on request from the corresponding author.

## Supplementary Materials

The following supporting information can be downloaded at: https://www.mdpi.com/article/10.3390/nu15194224/s1, Figure S1: Forest plots representing the risk of developing GDM; Figure S2: Forest plots representing the mean difference of fasting glucose; Figure S3: Forest plots representing the mean difference of 1h-OGTT; Figure S4: Forest plots representing the mean difference of 2h-OGTT; Figure S5: Forest plots representing the risk of insulin need; Figure S6: Forest plots representing the risk of pregnancy-induced hypertensive disorders; Figure S7: Forest plots representing the risk of preterm birth; Figure S8: Forest plots representing the mean difference of gestational age at birth; Figure S9: Forest plots representing the risk of C-section rate; Figure S10: Forest plots representing the risk of shoulder dystocia; Figure S11: Forest plots representing the mean difference of birthweight; Figure S12: Forest plots representing the risk of macrosomia; Figure S13: Forest plots representing the risk of neonatal hypoglycemia; Figure S14: Forest plots representing the risk of NICU admission; Table S1: PRISMA checklist; Table S2: Inclusion and exclusion criteria; Table S3: Risk of bias assessment using the Risk of Bias 2 tool; Table S4: GRADE: The quality of evidence in the inositol treated groups compared to placebo.

## Abbreviations

GDM	gestational diabetes mellitus
RCT	randomized controlled trial
OGTT	were oral glucose tolerance test
RR	risk ratio
MD	mean difference
CI	confidence interval
T2DM	type-2 diabetes mellitus
MI	myoinositol
DCI	D-chiro-inositol
GLUT4	glucose transporter type 4
PICO	population: intervention, comparator, outcome
LGA	large for gestational age
IUGR	intrauterine growth restriction
SD	standard deviation
RoB2	Risk-of-bias tool for randomized trials
GRADE	Grades of Recommendation, Assessment, Development, and Evaluation
REML	Restricted maximum likelihood
IADPSG	International Association of Diabetes and Pregnancy Study Groups
1 h-OGTT	one-hour glucose tolerance test
2 h-OGTT	two-hour glucose tolerance test
NICU	neonatal intensive care unit

## References

[1] American Diabetes Association (2003). Gestational Diabetes Mellitus. Diabetes Care.

[2] Hjort L., Novakovic B., Grunnet L.G., Maple-Brown L., Damm P., Desoye G., Saffery R. (2019). Diabetes in pregnancy and epigenetic mechanisms-how the first 9 months from conception might affect the child’s epigenome and later risk of disease. Lancet Diabetes Endocrinol..

[3] Hod M., Kapur A., Sacks D.A., Hadar E., Agarwal M., Di Renzo G.C., Roura L.C., McIntyre H.D., Morris J.L., Divakar H. (2015). The International Federation of Gynecology and Obstetrics (FIGO) Initiative on gestational diabetes mellitus: A pragmatic guide for diagnosis, management, and care. Int. J. Gynaecol. Obstet..

[4] Plows J.F., Reynolds C.M., Vickers M.H., Baker P.N., Stanley J.L. (2019). Nutritional Supplementation for the Prevention and/or Treatment of Gestational Diabetes Mellitus. Curr. Diabetes Rep..

[5] Kramer C.K., Campbell S., Retnakaran R. (2019). Gestational diabetes and the risk of cardiovascular disease in women: A systematic review and meta-analysis. Diabetologia.

[6] Vounzoulaki E., Khunti K., Abner S.C., Tan B.K., Davies M.J., Gillies C.L. (2020). Progression to type 2 diabetes in women with a known history of gestational diabetes: Systematic review and meta-analysis. BMJ.

[7] Quaresima P., Saccone G., Pellegrino R., Vaccarisi S., Taranto L., Mazzulla R., Bernardo S., Venturella R., Di Carlo C., Morelli M. (2021). Incidental diagnosis of a pancreatic adenocarcinoma in a woman affected by gestational diabetes mellitus: Case report and literature review. Am. J. Obstet. Gynecol. MFM.

[8] Kamińska K., Stenclik D., Błażejewska W., Bogdański P., Moszak M. (2022). Probiotics in the Prevention and Treatment of Gestational Diabetes Mellitus (GDM): A Review. Nutrients.

[9] Zhang D.Y., Cheng D.C., Cao Y.N., Su Y., Chen L., Liu W.Y., Yu Y.X., Xu X.M. (2022). The effect of dietary fiber supplement on prevention of gestational diabetes mellitus in women with pre-pregnancy overweight/obesity: A randomized controlled trial. Front. Pharmacol..

[10] Hashemi Tari S., Sohouli M.H., Lari A., Fatahi S., Rahideh S.T. (2021). The effect of inositol supplementation on blood pressure: A systematic review and meta-analysis of randomized-controlled trials. Clin. Nutr. ESPEN.

[11] Milewska E.M., Czyzyk A., Meczekalski B., Genazzani A.D. (2016). Inositol and human reproduction. From cellular metabolism to clinical use. Gynecol. Endocrinol..

[12] Qu X., Donnelly R. (2020). Sex Hormone-Binding Globulin (SHBG) as an Early Biomarker and Therapeutic Target in Polycystic Ovary Syndrome. Int. J. Mol. Sci..

[13] Greff D., Juhász A.E., Váncsa S., Váradi A., Sipos Z., Szinte J., Park S., Hegyi P., Nyirády P., Ács N. (2023). Inositol is an effective and safe treatment in polycystic ovary syndrome: A systematic review and meta-analysis of randomized controlled trials. Reprod. Biol. Endocrinol..

[14] Plows J.F., Stanley J.L., Baker P.N., Reynolds C.M., Vickers M.H. (2018). The Pathophysiology of Gestational Diabetes Mellitus. Int. J. Mol. Sci..

[15] Schneider S. (2015). Inositol transport proteins. FEBS Lett..

[16] Wei J., Yan J., Yang H. (2022). Inositol Nutritional Supplementation for the Prevention of Gestational Diabetes Mellitus: A Systematic Review and Meta-Analysis of Randomized Controlled Trials. Nutrients.

[17] Caputo M., Bona E., Leone I., Samà M.T., Nuzzo A., Ferrero A., Aimaretti G., Marzullo P., Prodam F. (2020). Inositols and metabolic disorders: From farm to bedside. J. Tradit. Complement. Med..

[18] Genazzani A.D., Santagni S., Rattighieri E., Chierchia E., Despini G., Marini G., Prati A., Simoncini T. (2014). Modulatory role of D-chiro-inositol (DCI) on LH and insulin secretion in obese PCOS patients. Gynecol. Endocrinol..

[19] Dinicola S., Unfer V., Facchinetti F., Soulage C.O., Greene N.D., Bizzarri M., Laganà A.S., Chan S.Y., Bevilacqua A., Pkhaladze L. (2021). Inositols: From Established Knowledge to Novel Approaches. Int. J. Mol. Sci..

[20] Page M.J., McKenzie J.E., Bossuyt P.M., Boutron I., Hoffmann T.C., Mulrow C.D., Shamseer L., Tetzlaff J.M., Akl E.A., Brennan S.E. (2021). The PRISMA 2020 statement: An updated guideline for reporting systematic reviews. BMJ.

[21] Higgins J.P.T., Thomas J., Chandler J., Cumpston M., Li T., Page M.J., Welch V.A. (2019). Cochrane Handbook for Systematic Reviews of Interventions.

[22] McHugh M.L. (2012). Interrater reliability: The kappa statistic. Biochem. Med..

[23] Sterne J.A.C., Savović J., Page M.J., Elbers R.G., Blencowe N.S., Boutron I., Cates C.J., Cheng H.Y., Corbett M.S., Eldridge S.M. (2019). RoB 2: A revised tool for assessing risk of bias in randomised trials. BMJ.

[24] Iorio A., Spencer F.A., Falavigna M., Alba C., Lang E., Burnand B., McGinn T., Hayden J., Williams K., Shea B. (2015). Use of GRADE for assessment of evidence about prognosis: Rating confidence in estimates of event rates in broad categories of patients. BMJ.

[25] Balduzzi S., Rücker G., Schwarzer G. (2019). How to perform a meta-analysis with R: A practical tutorial. Evid.-Based Ment. Health.

[26] Harrer M., Cuijpers P., Furukawa T., Ebert D.D. (2019). dmetar: Companion R Package For The Guide ‘Doing Meta-Analysis in R’.

[27] Viechtbauer W. (2010). Conducting Meta-Analyses in R with the metafor Package. J. Stat. Softw..

[28] Mantel N., Haenszel W. (1959). Statistical Aspects of the Analysis of Data From Retrospective Studies of Disease. JNCI J. Natl. Cancer Inst..

[29] Paule R.C., Mandel J. (1982). Consensus Values and Weighting Factors. J. Res. Natl. Bur. Stand..

[30] Thompson S.G., Turner R.M., Warn D.E. (2001). Multilevel models for meta-analysis, and their application to absolute risk differences. Stat. Methods Med. Res..

[31] Viechtbauer W. (2005). Bias and Efficiency of Meta-Analytic Variance Estimators in the Random-Effects Model. J. Educ. Behav. Stat..

[32] IntHout J., Ioannidis J.P.A., Rovers M.M., Goeman J.J. (2016). Plea for routinely presenting prediction intervals in meta-analysis. BMJ Open.

[33] Celentano C., Matarrelli B., Pavone G., Vitacolonna E., Mattei P.A., Berghella V., Liberati M. (2020). The influence of different inositol stereoisomers supplementation in pregnancy on maternal gestational diabetes mellitus and fetal outcomes in high-risk patients: A randomized controlled trial. J. Matern. Fetal Neonatal Med..

[34] D’Anna R., Di Benedetto A., Scilipoti A., Santamaria A., Interdonato M.L., Petrella E., Neri I., Pintaudi B., Corrado F., Facchinetti F. (2015). Myo-inositol Supplementation for Prevention of Gestational Diabetes in Obese Pregnant Women: A Randomized Controlled Trial. Obstet. Gynecol..

[35] D’Anna R., Scilipoti A., Giordano D., Caruso C., Cannata M.L., Interdonato M.L., Corrado F., Di Benedetto A. (2013). myo-Inositol supplementation and onset of gestational diabetes mellitus in pregnant women with a family history of type 2 diabetes: A prospective, randomized, placebo-controlled study. Diabetes Care.

[36] Esmaeilzadeh S., Ghadimi R., Mashayekh-Amiri S., Delavar M.A., Basirat Z. (2022). The effect of myo-inositol supplementation on the prevention of gestational diabetes in overweight pregnant women: A randomized, double-blind, controlled trial. Minerva Obstet. Gynecol..

[37] Farren M., Daly N., McKeating A., Kinsley B., Turner M.J., Daly S. (2017). The Prevention of Gestational Diabetes Mellitus with Antenatal Oral Inositol Supplementation: A Randomized Controlled Trial. Diabetes Care.

[38] Matarrelli B., Vitacolonna E., D’Angelo M., Pavone G., Mattei P.A., Liberati M., Celentano C. (2013). Effect of dietary myo-inositol supplementation in pregnancy on the incidence of maternal gestational diabetes mellitus and fetal outcomes: A randomized controlled trial. J. Matern. Fetal Neonatal Med..

[39] Santamaria A., Di Benedetto A., Petrella E., Pintaudi B., Corrado F., D’Anna R., Neri I., Facchinetti F. (2016). Myo-inositol may prevent gestational diabetes onset in overweight women: A randomized, controlled trial. J. Matern. Fetal Neonatal Med..

[40] Vitale S.G., Corrado F., Caruso S., Di Benedetto A., Giunta L., Cianci A., D’Anna R. (2021). Myo-inositol supplementation to prevent gestational diabetes in overweight non-obese women: Bioelectrical impedance analysis, metabolic aspects, obstetric and neonatal outcomes—A randomized and open-label, placebo-controlled clinical trial. Int. J. Food Sci. Nutr..

[41] Balsells M., García-Patterson A., Gich I., Corcoy R. (2009). Maternal and Fetal Outcome in Women with Type 2 versus Type 1 Diabetes Mellitus: A Systematic Review and Metaanalysis. J. Clin. Endocrinol. Metab..

[42] Clausen T.D., Mathiesen E., Ekbom P., Hellmuth E., Mandrup-Poulsen T., Damm P. (2005). Poor Pregnancy Outcome in Women with Type 2 Diabetes. Diabetes Care.

[43] Juan J., Sun Y., Wei Y., Wang S., Song G., Yan J., Zhou P., Yang H. (2022). Progression to type 2 diabetes mellitus after gestational diabetes mellitus diagnosed by IADPSG criteria: Systematic review and meta-analysis. Front. Endocrinol..

[44] Scholtens D.M., Kuang A., Lowe L.P., Hamilton J., Lawrence J.M., Lebenthal Y., Brickman W.J., Clayton P., Ma R.C., McCance D. (2019). Hyperglycemia and Adverse Pregnancy Outcome Follow-up Study (HAPO FUS): Maternal Glycemia and Childhood Glucose Metabolism. Diabetes Care.

[45] Facchinetti F., Unfer V., Dewailly D., Kamenov Z.A., Diamanti-Kandarakis E., Laganà A.S., Nestler J.E., Soulage C.O. (2020). Inositols in Polycystic Ovary Syndrome: An Overview on the Advances. Trends Endocrinol. Metab..

[46] Unfer V., Nestler J.E., Kamenov Z.A., Prapas N., Facchinetti F. (2016). Effects of Inositol(s) in Women with PCOS: A Systematic Review of Randomized Controlled Trials. Int. J. Endocrinol..

[47] Chan K.Y., Wong M.M.H., Pang S.S.H., Lo K.K.H. (2021). Dietary supplementation for gestational diabetes prevention and management: A meta-analysis of randomized controlled trials. Arch. Gynecol. Obstet..

[48] Guo X., Guo S., Miao Z., Li Z., Zhang H. (2018). Myo-inositol lowers the risk of developing gestational diabetic mellitus in pregnancies: A systematic review and meta-analysis of randomized controlled trials with trial sequential analysis. J. Diabetes Its Complicat..

[49] Motuhifonua S.K., Lin L., Alsweiler J., Crawford T.J., Crowther C.A. (2023). Antenatal dietary supplementation with myo-inositol for preventing gestational diabetes. Cochrane Database Syst. Rev..

[50] Vitagliano A., Saccone G., Cosmi E., Visentin S., Dessole F., Ambrosini G., Berghella V. (2019). Inositol for the prevention of gestational diabetes: A systematic review and meta-analysis of randomized controlled trials. Arch. Gynecol. Obstet..

[51] Oostdam N., van Poppel M.N., Wouters M.G., van Mechelen W. (2011). Interventions for preventing gestational diabetes mellitus: A systematic review and meta-analysis. J. Women’s Health.

[52] Dell’Edera D., Sarlo F., Allegretti A., Simone F., Lupo M.G., Epifania A.A. (2017). The influence of D-chiro-inositol and D-myo-inositol in pregnant women with glucose intolerance. Biomed. Rep..

[53] Godfrey K.M., Barton S.J., El-Heis S., Kenealy T., Nield H., Baker P.N., Chong Y.S., Cutfield W., Chan S.-Y., Group N.S. (2021). Myo-Inositol, Probiotics, and Micronutrient Supplementation From Preconception for Glycemia in Pregnancy: NiPPeR International Multicenter Double-Blind Randomized Controlled Trial. Diabetes Care.

[54] Saravanan P., Sukumar N., Adaikalakoteswari A., Goljan I., Venkataraman H., Gopinath A., Bagias C., Yajnik C.S., Stallard N., Ghebremichael-Weldeselassie Y. (2021). Association of maternal vitamin B12 and folate levels in early pregnancy with gestational diabetes: A prospective UK cohort study (PRiDE study). Diabetologia.

[55] D’Anna R., Corrado F., Loddo S., Gullo G., Giunta L., Di Benedetto A. (2021). Myoinositol plus α-lactalbumin supplementation, insulin resistance and birth outcomes in women with gestational diabetes mellitus: A randomized, controlled study. Sci. Rep..

[56] Fraticelli F., Celentano C., Zecca I.A., Di Vieste G., Pintaudi B., Liberati M., Franzago M., Di Nicola M., Vitacolonna E. (2018). Effect of inositol stereoisomers at different dosages in gestational diabetes: An open-label, parallel, randomized controlled trial. Acta Diabetol..

[57] Hegyi P., Erőss B., Izbéki F., Párniczky A., Szentesi A. (2021). Accelerating the translational medicine cycle: The Academia Europaea pilot. Nat. Med..

[58] Hegyi P., Petersen O.H., Holgate S., Erőss B., Garami A., Szakács Z., Dobszai D., Balaskó M., Kemény L., Peng S. (2020). Academia Europaea Position Paper on Translational Medicine: The Cycle Model for Translating Scientific Results into Community Benefits. J. Clin. Med..

[59] Tahir F., Majid Z. (2019). Inositol Supplementation in the Prevention of Gestational Diabetes Mellitus. Cureus.

