# Supplementary material for: Myoinositols Prevent Gestational Diabetes Mellitus and Related Complications: A Systematic Review and Meta-Analysis of Randomized Controlled Trials
Source: Nutrients. 2023 Sep 30;15(19):4224. doi: 10.3390/nu15194224 (PMC10574514; doi:10.3390/nu15194224)
Supplement: Supplementary file 1 [file nutrients-15-04224-s001.zip › nutrients-2607887-supplementary.pdf]

Myoinositols Prevent Gestational Diabetes Mellitus and Related Complications: A Systematic Review and Meta-Analysis of Randomized Controlled Trials

Dorina Greff [1,2,3], Szilárd Váncsa [1,4,5], Alex Váradi [4,6,7], Julia Szinte [1,2,3], Sunjune Park [1,3] and Péter Hegyi [1,4,5], Péter Nyirády [1,8], Nándor Ács [1,2], Eszter Mária Horváth [3,*] and Szabolcs Várbíró [2,9,10]

1 Centre for Translational Medicine, Semmelweis University, 1085 Budapest, Hungary; acs.nandor@med.semmelweis-univ.hu (N.Á.)
2 Department of Obstetrics and Gynecology, Semmelweis University, 1083 Budapest, Hungary
3 Department of Physiology, Semmelweis University, Tűzoltó Str. 37-47, 1094 Budapest, Hungary
4 Institute for Translational Medicine, Medical School, University of Pécs, 7621 Pécs, Hungary
5 Institute of Pancreatic Diseases, Semmelweis University, 1085 Budapest, Hungary
6 Department of Metagenomics, University of Debrecen, 4032 Debrecen, Hungary
7 Department of Laboratory Medicine, Medical School, University of Pécs, 7621 Pécs, Hungary
8 Department of Urology, Semmelweis University, 1083 Budapest, Hungary
9 Workgroup for Science Management, Doctoral School, Semmelweis University, 1085 Budapest, Hungary
10 Department of Obstetrics and Gynecology, University of Szeged, 6725 Szeged, Hungary
* Correspondence: horvath.eszter@med.semmelweis-univ.hu; Tel.: +36-(1)-459-1500

TABLE OF CONTENT

Figure S1. Forest plots representing the risk of developing GDM

Figure S2. Forest plots representing the mean difference of fasting glucose

Figure S3. Forest plots representing the mean difference of 1h-OGTT

Figure S4. Forest plots representing the mean difference of 2h-OGTT

Figure S5. Forest plots representing the risk of insulin need

Figure S6. Forest plots representing the risk of pregnancy-induced hypertensive disorders

Figure S7. Forest plots representing the risk of preterm birth

Figure S8. Forest plots representing the mean difference of gestational age at birth

Figure S9. Forest plots representing the risk of C-section rate

Figure S10. Forest plots representing the risk of shoulder dystocia

Figure S11 Forest plots representing the mean difference of birthweight

Figure S12. Forest plots representing the risk of macrosomia

Figure S13. Forest plots representing the risk of neonatal hypoglycemia

Figure S14. Forest plots representing the risk of NICU admission

Table S1. PRISMA checklist

Table S2. Inclusion and exclusion criteria

Table S3. Risk of bias assessment using the Risk of Bias 2 tool

Table S4. GRADE: The quality of evidence in the inositol treated groups compared to placebo.

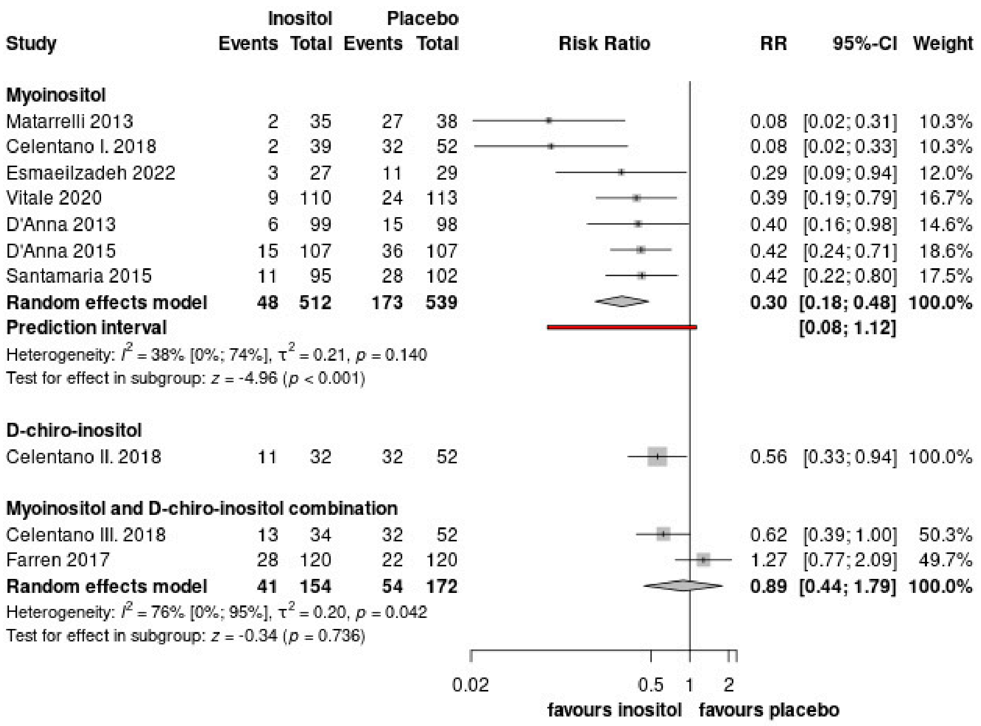

Figure S1. Forest plots representing the risk of developing GDM

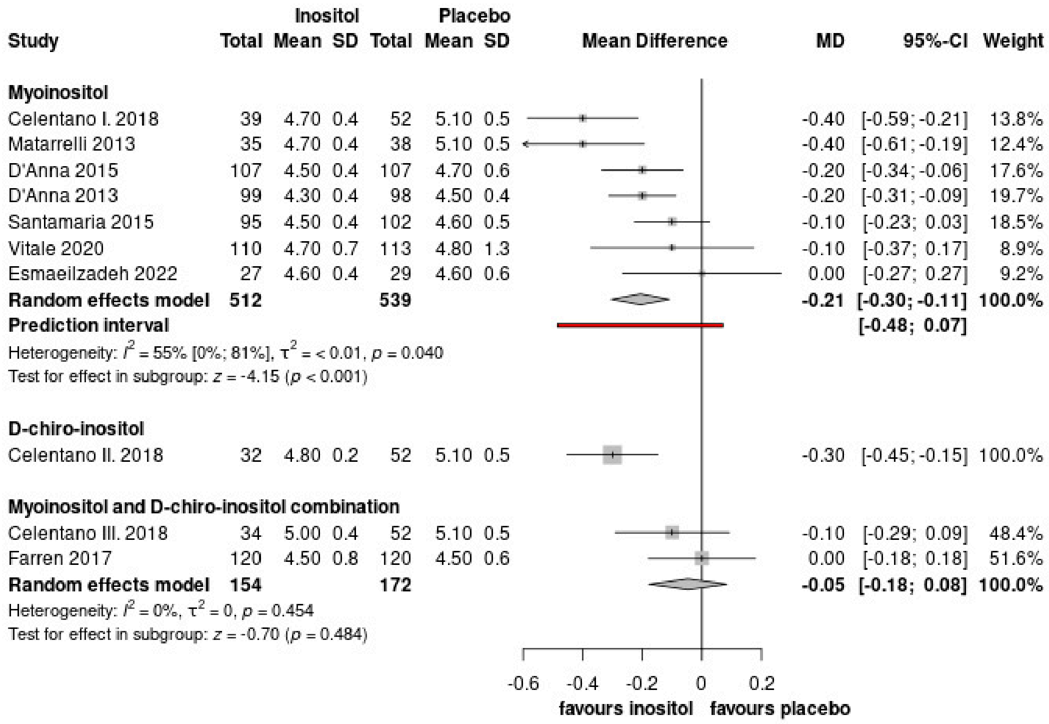

Figure S2. Forest plots representing the mean differences of fasting glucose

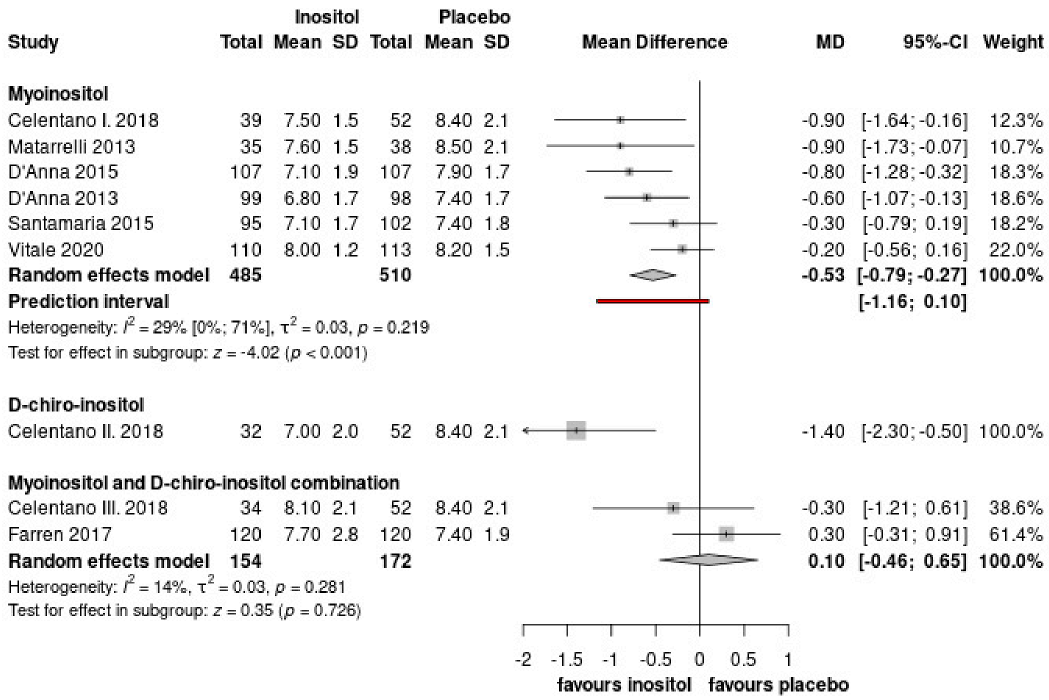

Figure S3. Forest plots representing the mean differences of 1h-OGTT

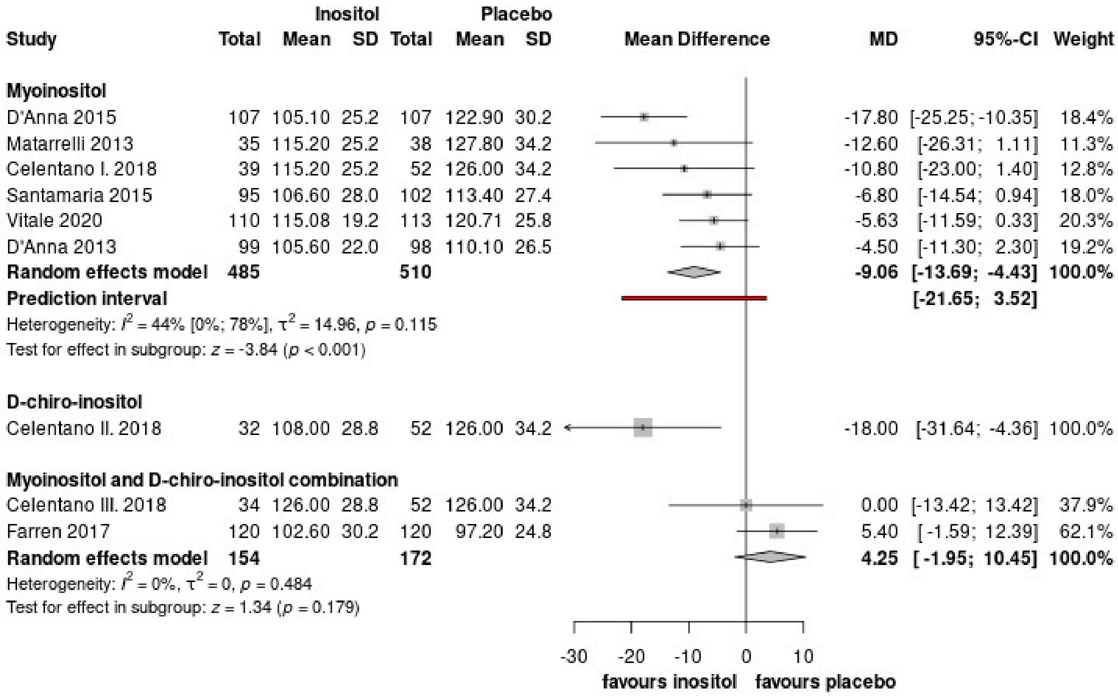

Figure S4 Forest plots representing the mean differences of 2h-OGTT

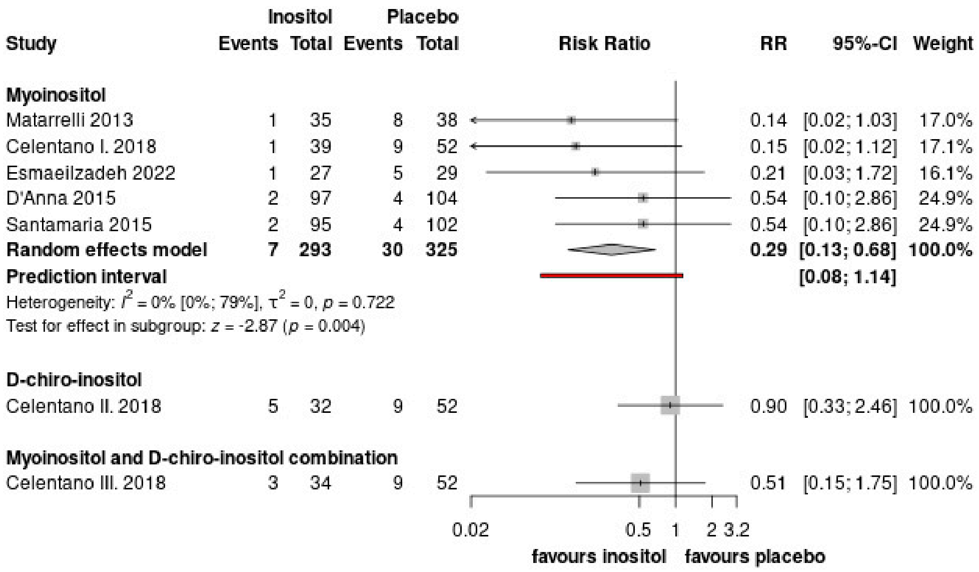

Figure S5. Forest plots representing the risk of insulin need

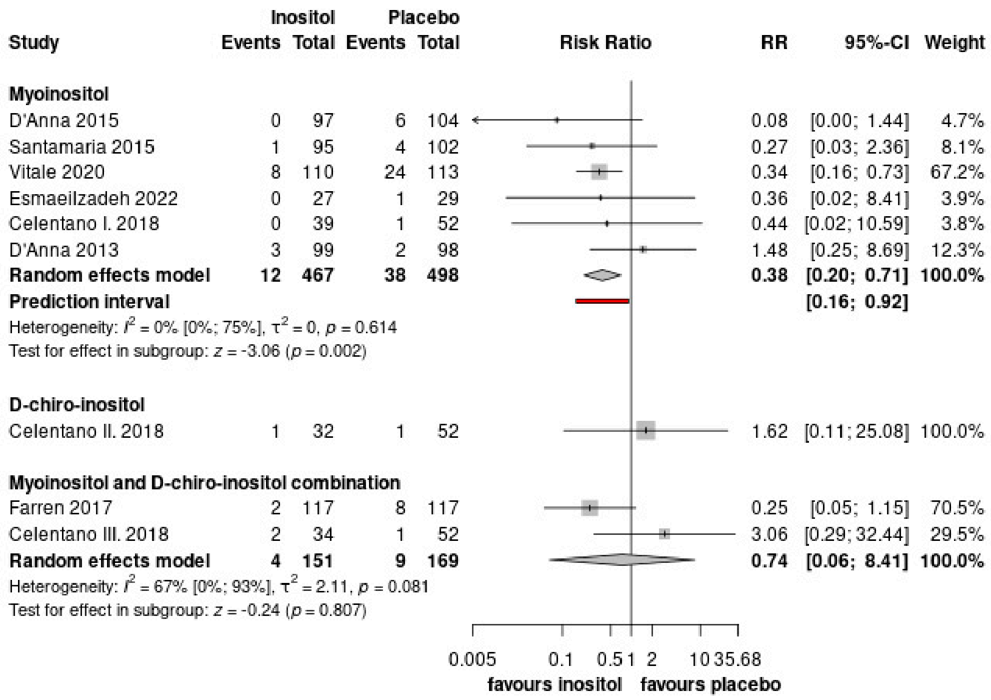

Figure S6. Forest plots representing the risk of pregnancy-induced hypertensive disorders

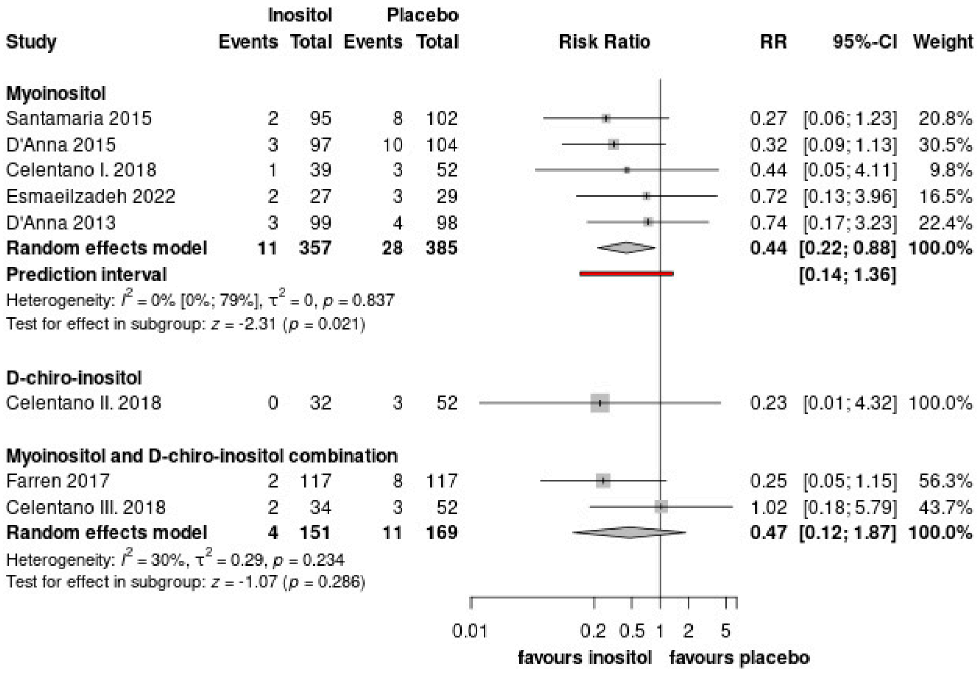

Figure S7. Forest plots representing the risk of preterm birth

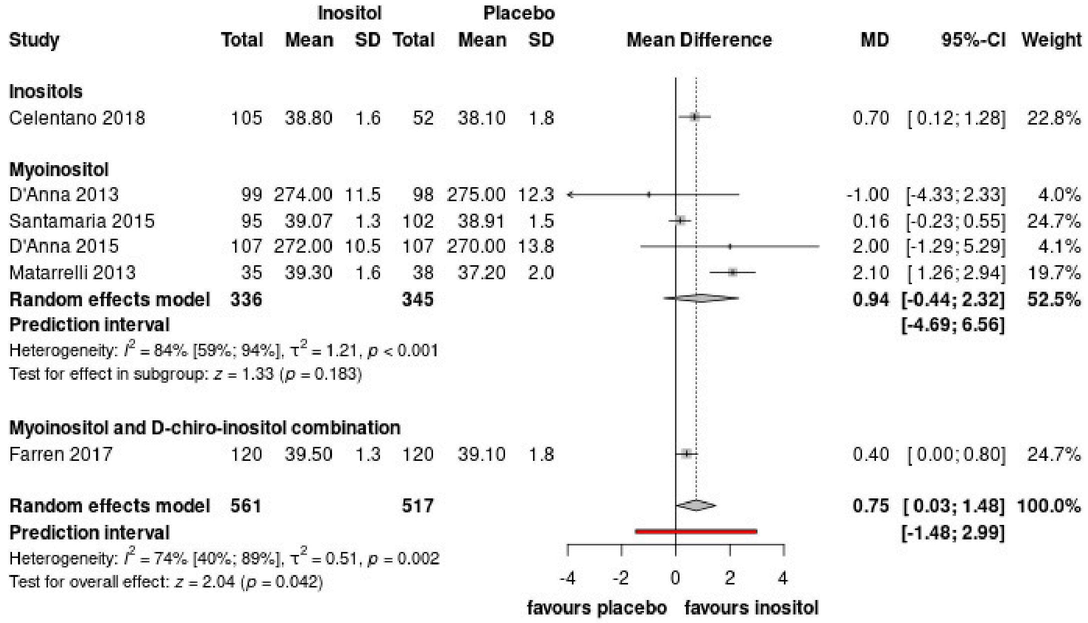

Figure S8a. Forest plots representing the mean difference of gestational age at birth

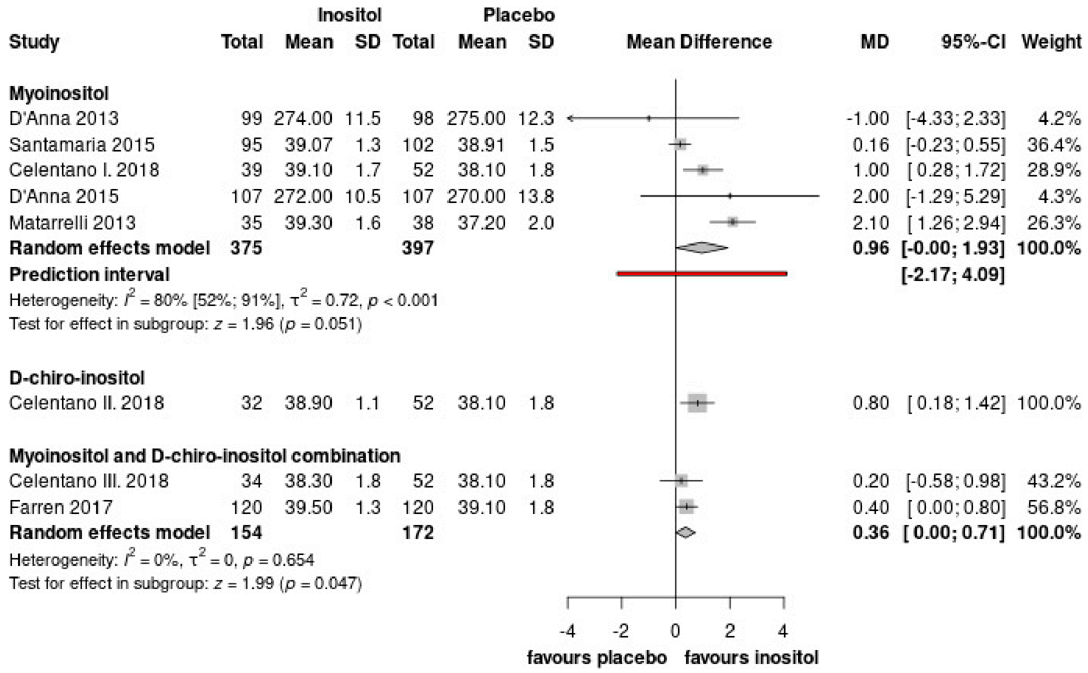

Figure S8b. Forest plots representing the mean difference of gestational age at birth

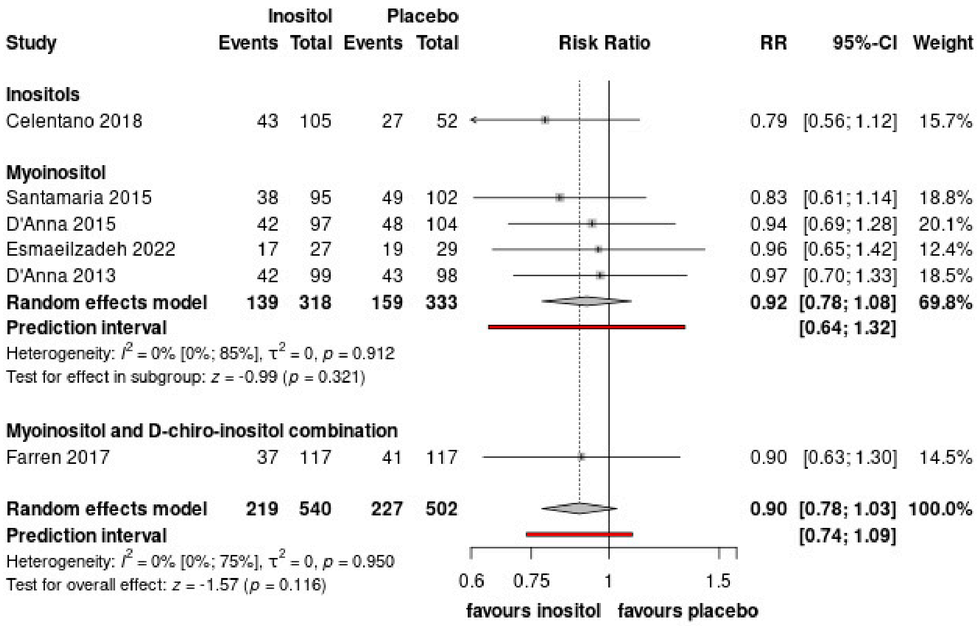

Figure S9a. Forest plots representing the risk of C-section rate

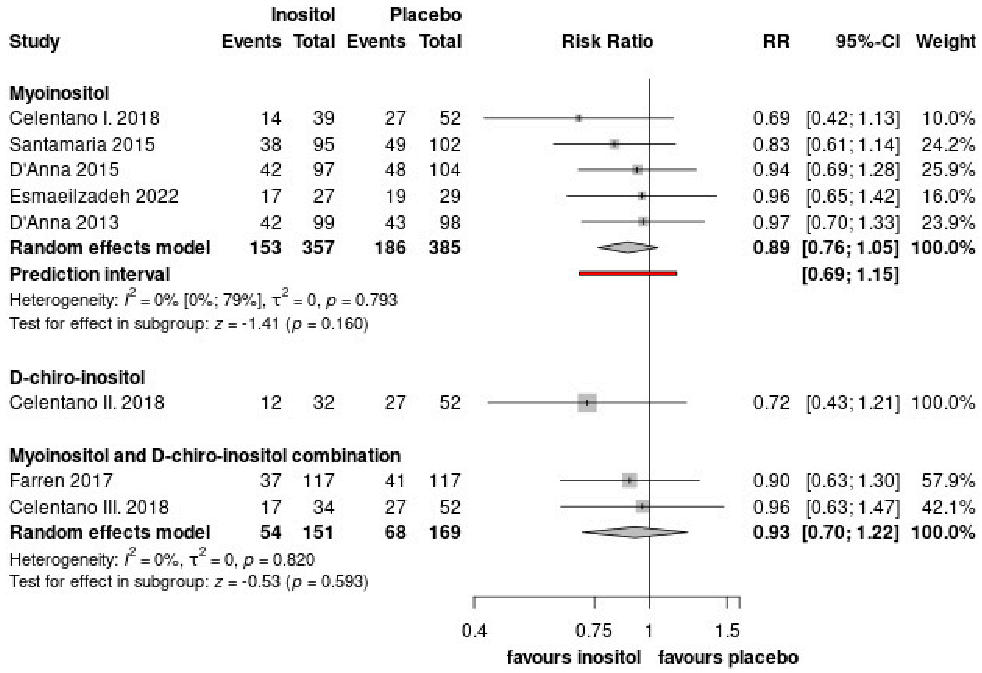

Figure S9b. Forest plots representing the risk of C-section rate

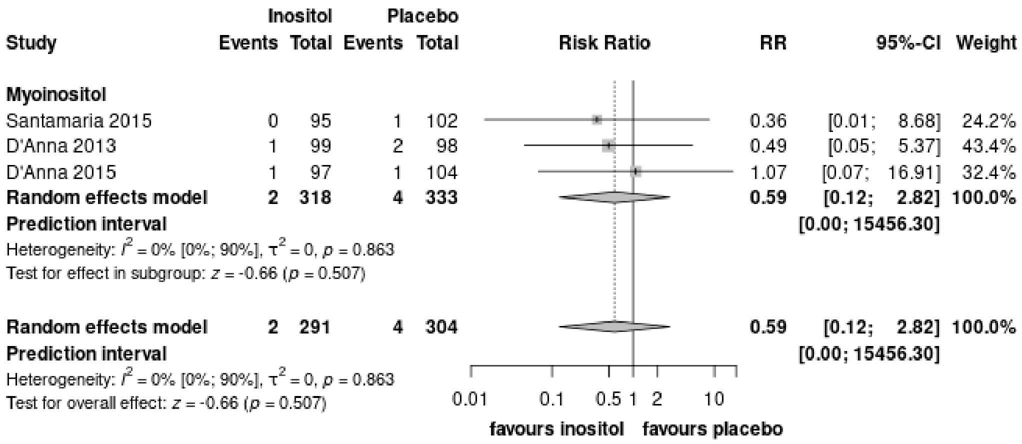

Figure S10. Forest plots representing the risk of shoulder dystocia

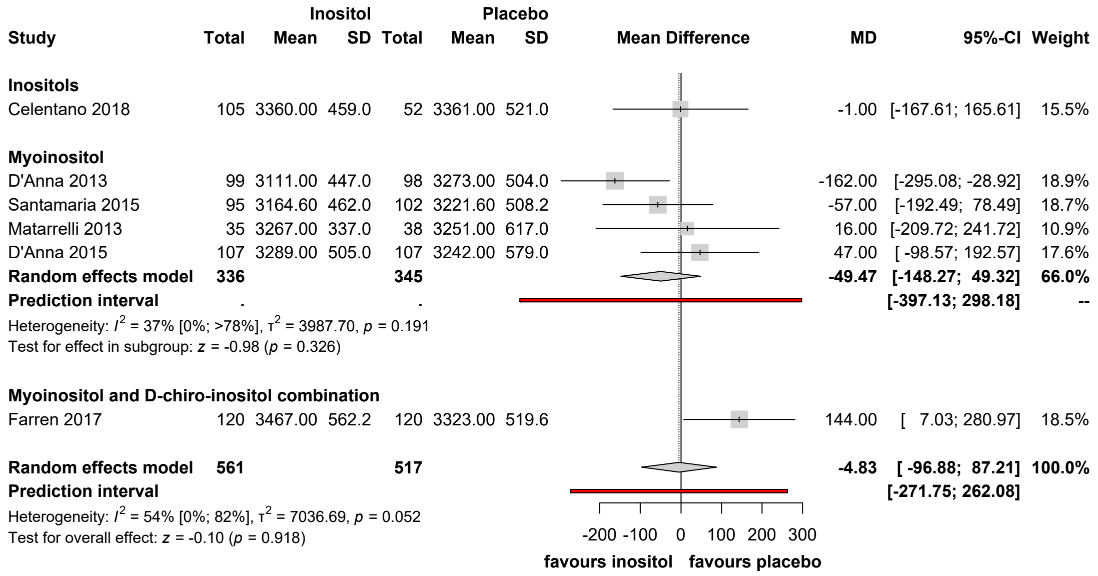

Figure S11a. Forest plots representing the mean difference of birthweight

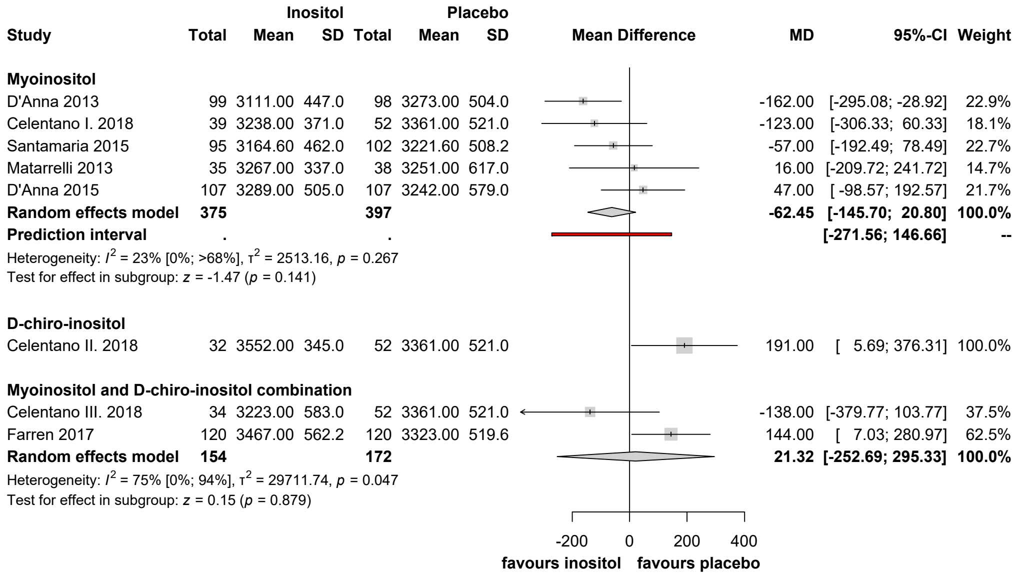

Figure S11b. Forest plots representing the mean difference of birthweight

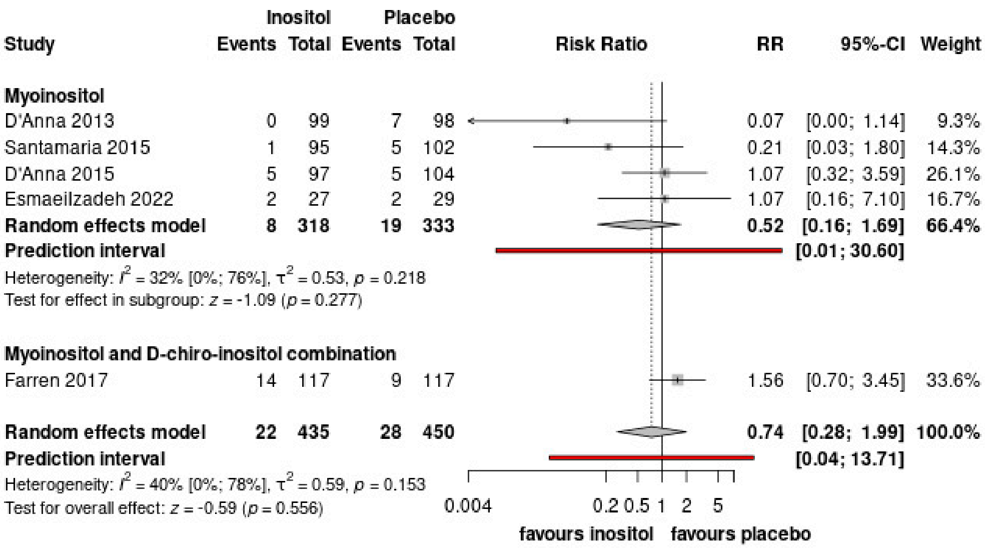

Figure S12. Forest plots representing the risk of macrosomia

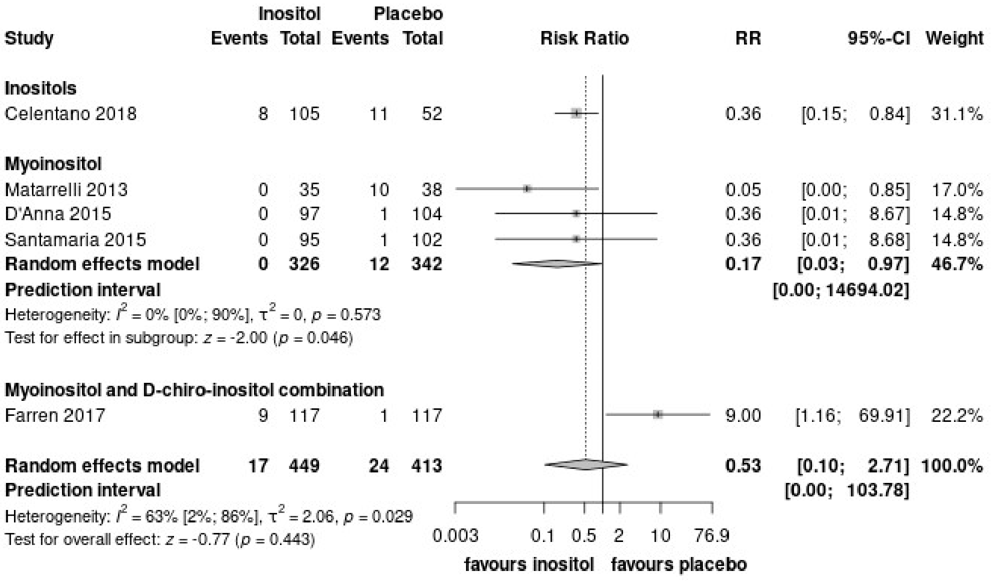

Figure S13. Forest plots representing the risk of neonatal hypoglycemia

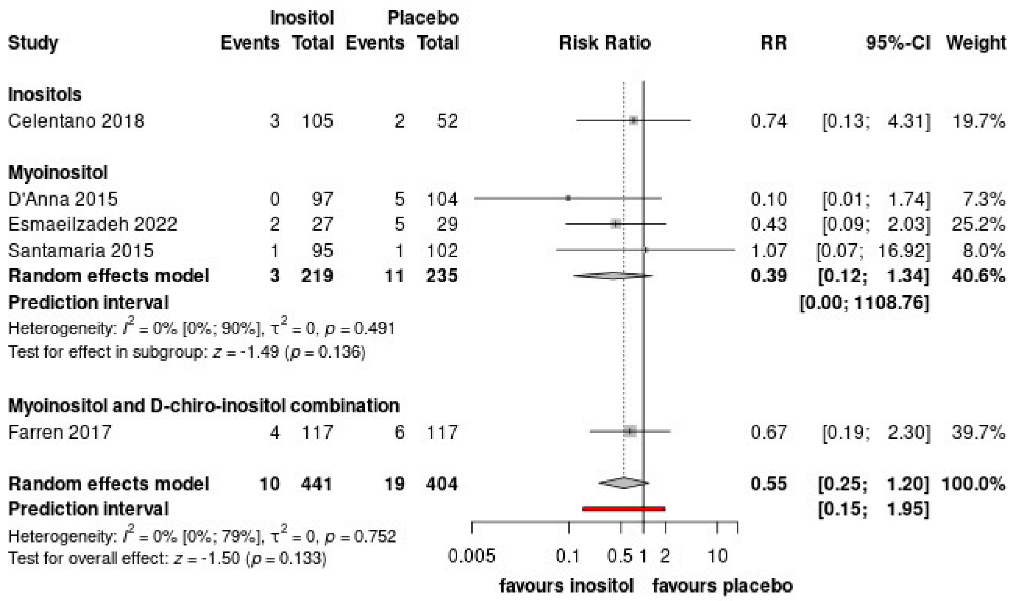

Figure S14a. Forest plots representing the risk of NICU admission

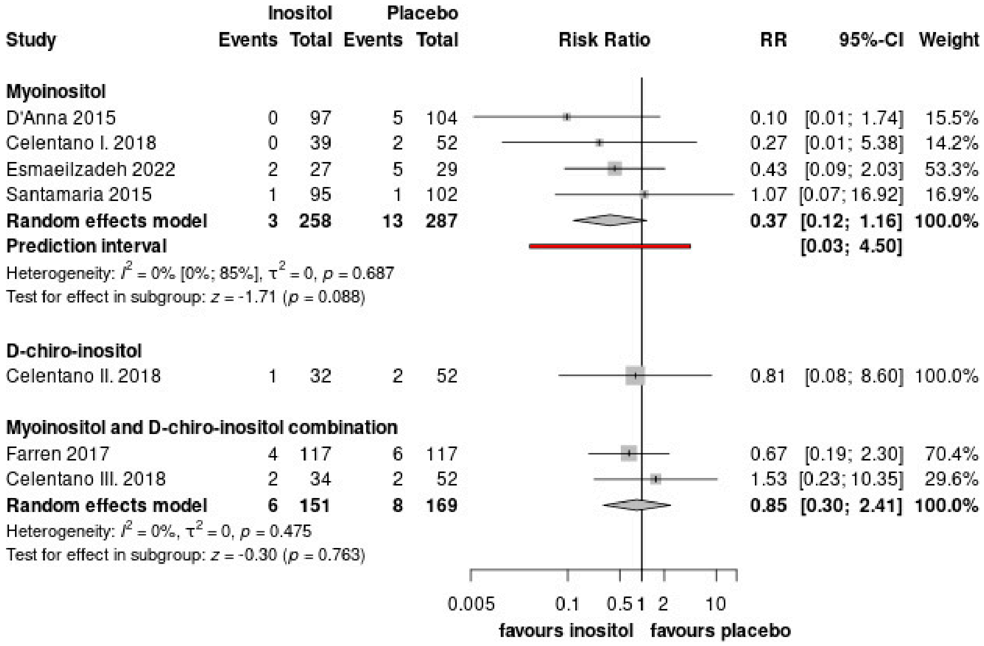

Figure S14b. Forest plots representing the risk of NICU admission

Table S1. PRISMA checklist

Section and topic	Item #	Checklist item	Location where item is reported
**Title**			
Title	1	Identify the report as a systematic review.	2-3
**Abstract**			
Abstract	2	See the PRISMA 2020 for Abstracts checklist (table 2).	
**Introduction**			
Rationale	3	Describe the rationale for the review in the context of existing knowledge.	81-106
Objectives	4	Provide an explicit statement of the objective(s) or question(s) the review addresses.	107-110
**Methods**			
Eligibility criteria	5	Specify the inclusion and exclusion criteria for the review and how studies were grouped for the syntheses.	107-112
Information sources	6	Specify all databases, registers, websites, organisations, reference lists and other sources searched or consulted to identify studies. Specify the date when each source was last searched or consulted.	134-140
Search strategy	7	Present the full search strategies for all databases, registers and websites, including any filters and limits used.	134-140
Selection process	8	Specify the methods used to decide whether a study met the inclusion criteria of the review, including how many reviewers screened each record and each report retrieved, whether they worked independently, and if applicable, details of automation tools used in the process.	142-145
Data collection process	9	Specify the methods used to collect data from reports, including how many reviewers collected data from each report, whether they worked independently, any processes for obtaining or confirming data from study investigators, and if applicable, details of automation tools used in the process.	145-156
Data items	10a	List and define all outcomes for which data were sought. Specify whether all results that were compatible with each outcome domain in each study were sought (e.g. for all measures, time points, analyses), and if not, the methods used to decide which results to collect.	123-132
	10b	List and define all other variables for which data were sought (e.g. participant and intervention characteristics, funding sources). Describe any assumptions made about any missing or unclear information.	147-156

Study risk of bias assessment	11	Specify the methods used to assess risk of bias in the included studies, including details of the tool(s) used, how many reviewers assessed each study and whether they worked independently, and if applicable, details of automation tools used in the process.	158-161
Effect measures	12	Specify for each outcome the effect measure(s) (e.g. risk ratio, mean difference) used in the synthesis or presentation of results.	168-172
Synthesis methods	13a	Describe the processes used to decide which studies were eligible for each synthesis (e.g. tabulating the study intervention characteristics and comparing against the planned groups for each synthesis (item #5)).	166-180
	13b	Describe any methods required to prepare the data for presentation or synthesis, such as handling of missing summary statistics, or data conversions.	166-180
	13c	Describe any methods used to tabulate or visually display results of individual studies and syntheses.	166-180
	13d	Describe any methods used to synthesise results and provide a rationale for the choice(s). If meta-analysis was performed, describe the model(s), method(s) to identify the presence and extent of statistical heterogeneity, and software package(s) used.	166-180
	13e	Describe any methods used to explore possible causes of heterogeneity among study results (e.g. subgroup analysis, meta-regression).	166-180
	13f	Describe any sensitivity analyses conducted to assess robustness of the synthesised results.	-
Reporting bias assessment	14	Describe any methods used to assess risk of bias due to missing results in a synthesis (arising from reporting biases).	157-161
Certainty assessment	15	Describe any methods used to assess certainty (or confidence) in the body of evidence for an outcome.	162-164
**Results**			
Study selection	16a	Describe the results of the search and selection process, from the number of records identified in the search to the number of studies included in the review, ideally using a flow diagram (see fig 1).	184-186
	16b	Cite studies that might appear to meet the inclusion criteria, but which were excluded, and explain why they were excluded.	182-184
Study characteristics	17	Cite each included study and present its characteristics.	198-201
Risk of bias in studies	18	Present assessments of risk of bias for each included study.	242-247
Results of individual studies	19	For all outcomes, present, for each study: (a) summary statistics for each group (where appropriate) and (b) an effect estimate and its precision (e.g. confidence/credible interval), ideally using structured tables or plots.	204-241
	20a	For each synthesis, briefly summarise the characteristics and risk of bias among contributing studies.	

Results of syntheses	20b	Present results of all statistical syntheses conducted. If meta-analysis was done, present for each the summary estimate and its precision (e.g. confidence/credible interval) and measures of statistical heterogeneity. If comparing groups, describe the direction of the effect.	203-241
	20c	Present results of all investigations of possible causes of heterogeneity among study results.	203-241
	20d	Present results of all sensitivity analyses conducted to assess the robustness of the synthesised results.	203-241
Reporting biases	21	Present assessments of risk of bias due to missing results (arising from reporting biases) for each synthesis assessed.	242-247
Certainty of evidence	22	Present assessments of certainty (or confidence) in the body of evidence for each outcome assessed.	248-249
**Discussion**			
Discussion	23a	Provide a general interpretation of the results in the context of other evidence.	251-321
	23b	Discuss any limitations of the evidence included in the review.	323-330
	23c	Discuss any limitations of the review processes used.	323-330
	23d	Discuss implications of the results for practice, policy, and future research.	332-340
**Other information**			
Registration and protocol	24a	Provide registration information for the review, including register name and registration number, or state that the review was not registered.	114
	24b	Indicate where the review protocol can be accessed, or state that a protocol was not prepared.	114
	24c	Describe and explain any amendments to information provided at registration or in the protocol.	-
Support	25	Describe sources of financial or non-financial support for the review, and the role of the funders or sponsors in the review.	26-30
Competing interests	26	Declare any competing interests of review authors.	25
Availability of data, code, and other materials	27	Report which of the following are publicly available and where they can be found: template data collection forms; data extracted from included studies; data used for all analyses; analytic code; any other materials used in the review.	347-349

Table S2. Inclusion and exclusion criteria

Author	Inclusion criteria	Exclusion criteria
Celentano, 2018 (1)	„Consecutive singleton pregnant women attending our High-Risk Pregnancy Unit of the Hospital of University of "G. d'Annunzio" in Chieti from January 2012 to July 2017 upon referral for an elevated fasting glucose (glycemia >5.1 mmol/L or 92 mg/dL and <7.0 mmol/L or 126 mg/dL) at first trimester blood exams according to National Guidelines [17] were enrolled during their first visit."	„Pregestational obesity (BMI above 35), patients younger than 18 years-old, multiple gestations, and pregestational diabetes were exclusion criteria."
D'Anna, 2013 (2)	„1) first-degree relatives (mother father, or both) affected by type 2 diabetes, 2) prepregnancy BMI ,30 kg/m2, 3) fasting plasma glucose ,126 mg/dL and random glycemia ,200 mg/dL, 4) single pregnancy, and 5) Caucasian race."	„Exclusion criteria were as followings: 1) prepregnancy BMI >30 kg/m2, 2) previous GDM, 3) pregestational diabetes, 4) firsttrimester glicosuria, 5) first-degree relative(s) (mother or father) not affected by type 2 diabetes, 6) fasting plasma glucose >126 mg/dL or random glycemia >200 mg/dL, 7) twin pregnancies, 8) associated therapy with corticosteroids, 9) not Caucasian race, and 10) PCOS women."
D'Anna, 2015 (3)	„Inclusion criteria were 1) prepregnancy body mass index (BMI) (calculated as weight (kg)/m2) 30 or greater and 2) singleton gestation."	Exclusion criteria were 1) previous GDM, 2) pregestational diabetes, 3) first-trimester glycosuria (urine glucose value 10 mg/dL or greater), 4) first-trimester fasting plasma glucose 126 mg/dL or greater or random plasma glucose 200 mg/dL or greater, 5) concomitant treatment with corticosteroids; and 6) hypertension or renal or hepatic disease.
Esmaeilzadeh, 2022 (4)	"Overweight, pregnant women (prepregnancy BMI ≥ 25 and < 30 kg/ m2), aged 18–40 "	"The women with diabetes, a history of hypertension, cardiovascular diseases, current smoking or drinking habits were excluded from the study. Those who had experienced the death of family members or received corticosteroids during pregnancy

		were also excluded from the study.”
Farren, 2017 (5)	„Women with a family history in a first-degree relative of diabetes, either type 1 or type 2, were eligible for inclusion.”	„Exclusion criteria were: 1) age younger than 18 years, 2) multiple pregnancy, 3) limited understanding of English, and 4) any pre-existing liver or kidney disease or diabetes.”
Matarelli, 2013 (6)	„Consecutive singleton pregnant women attending our High Risk Pregnancy Unit of the Hospital of University ‘‘Gabriele d’Annunzio’’ in Chieti from August 2010 to April 2011 upon referral for an elevated fasting glucose (glycemia >5.1 mmol/L or 92 mg/dL and <7.0 mmol/L or 126 mg/dL) according to National Guidelines were eligible for enrollment.”	„Pre-gestational obesity (BMI above 35) and refusal to participate were the only exclusion criteria.”
Santamaria, 2016 (7)	„The inclusion criteria were: (1) pre-pregnancy BMI >25 and 530 kg/m2, (2) first trimester fasting plasma glucose <126 mg/dl and/or random glycemia<200 mg/dl, (3) single pregnancy and (4) Caucasian ethnicity.”	„Exclusion criteria were as follow: (1) pre-pregnancy BMI<25 and >30 kg/m2, (2) previous GDM, (3) pre-gestational diabetes, (4) first trimester glycosuria and (5) treatment with corticosteroids.”
Vitale, 2020 (8)	„… pre-pregnancy BMI > 25 and <30 kg/m2, first-trimester fasting plasma glucose <126 mg/dl and/or random glycaemia <200 mg/ dl, single pregnancy, and Caucasian ethnicity.”	„We excluded women who had a pre-pregnancy BMI <25 and > 30 kg/m2, previous GDM, pre-gestational diabetes, first-trimester glycosuria, and in treatment with corticosteroids.”

Table S3. Risk of bias assessment using the Risk of Bias 2 tool

Study ID	Outcome	Randomization process	Deviations from intended interventions	Missing outcome data	Measurement of the outcome	Selection of the reported result	Overall
Celentano	GDM	Low	Some concerns	Low	Low	Some concerns	Some concerns
Celentano	Insulin therapy	Low	Some concerns	Low	Low	Some concerns	Some concerns
Celentano	C-section rate	Low	Some concerns	Low	Low	Some concerns	Some concerns
Celentano	pregnancy-induced hypertensive disorders	Low	Some concerns	Low	Low	Some concerns	Some concerns
Celentano	Preterm birth	Low	Some concerns	Low	Low	Some concerns	Some concerns
Celentano	Neonatal hypoglycemia	Low	Some concerns	Low	Low	Some concerns	Some concerns
Celentano	Neonatal intensive care unit admission	Low	Some concerns	Low	Low	Some concerns	Some concerns
Celentano	OGTT 0'	Low	Some concerns	Low	Low	Some concerns	Some concerns
Celentano	OGTT 60'	Low	Some concerns	Low	Low	Some concerns	Some concerns
Celentano	OGTT 120'	Low	Some concerns	Low	Low	Some concerns	Some concerns
Celentano	Gestational age at birth	Low	Some concerns	Low	Low	Some concerns	Some concerns
Celentano	Birthweight	Low	Some concerns	Low	Low	Some concerns	Some concerns
Celentano	LGA	Low	Some concerns	Low	Low	Some concerns	Some concerns
D'Anna 2015	OGTT 0'	Some concerns	Some concerns	Some concerns	Low	Low	Some concerns
D'Anna 2015	OGTT 60'	Some concerns	Some concerns	Some concerns	Low	Low	Some concerns
D'Anna 2015	OGTT 120'	Some concerns	Some concerns	Some concerns	Low	Low	Some concerns
D'Anna 2015	GDM	Some concerns	Some concerns	Some concerns	Low	Low	Some concerns
D'Anna 2015	Fasting insulin	Some concerns	Some concerns	Some concerns	Low	Low	Some concerns
D'Anna 2015	Gestational age at birth	Some concerns	Some concerns	Some concerns	Low	Low	Some concerns
D'Anna 2015	Birth weight	Some concerns	Some concerns	Some concerns	Low	Low	Some concerns

D'Anna 2015	Macrosomia	Some concerns	Some concerns	Some concerns	Low	Low	Some concerns
D'Anna 2015	C-section rate	Some concerns	Some concerns	Some concerns	Low	Low	Some concerns
D'Anna 2015	pregnancy-induced hypertensive disorders	Some concerns	Some concerns	Some concerns	Low	Low	Some concerns
D'Anna 2015	Insulin therapy	Some concerns	Some concerns	Some concerns	Low	Low	Some concerns
D'Anna 2015	Neonatal hypoglycemia	Some concerns	Some concerns	Some concerns	Low	Low	Some concerns
D'Anna 2015	Preterm birth	Some concerns	Some concerns	Some concerns	Low	Low	Some concerns
D'Anna 2015	Shoulder dystocia	Some concerns	Some concerns	Some concerns	Low	Low	Some concerns
D'Anna 2015	Neonatal intensive care unit admission	Some concerns	Some concerns	Some concerns	Low	Low	Some concerns
D'Anna 2013	OGTT 0'	Low	Some concerns	Low	Low	Low	Some concerns
D'Anna 2013	OGTT 60'	Low	Some concerns	Low	Low	Low	Some concerns
D'Anna 2013	OGTT 120'	Low	Some concerns	Low	Low	Low	Some concerns
D'Anna 2013	Gestational age at birth	Low	Some concerns	Low	Low	Low	Some concerns
D'Anna 2013	Birth weight	Low	Some concerns	Low	Low	Low	Some concerns
D'Anna 2013	Macrosomia	Low	Some concerns	Low	Low	Low	Some concerns
D'Anna 2013	C-section rate	Low	Some concerns	Low	Low	Low	Some concerns
D'Anna 2013	pregnancy-induced hypertensive disorders	Low	Some concerns	Low	Low	Low	Some concerns
D'Anna 2013	Preterm birth	Low	Some concerns	Low	Low	Low	Some concerns
D'Anna 2013	Shoulder dystocia	Low	Some concerns	Low	Low	Low	Some concerns
D'Anna 2013	Neonatal hypoglycemia	Low	Some concerns	Low	Low	Low	Some concerns
D'Anna 2013	GDM	Low	Some concerns	Low	Low	Low	Some concerns

Esmaeilzadeh	GDM	Low	Low	Low	Low	Low	Low
Esmaeilzadeh	Insulin therapy	Low	Low	Low	Low	Low	Low
Esmaeilzadeh	C-section	Low	Low	Low	Low	Low	Low
Esmaeilzadeh	pregnancy-induced hypertensive disorders	Low	Low	Low	Low	Low	Low
Esmaeilzadeh	Macrosomia	Low	Low	Low	Low	Low	Low
Esmaeilzadeh	Preterm birth	Low	Low	Low	Low	Low	Low
Esmaeilzadeh	Shoulder dystocia	Low	Low	Low	Low	Low	Low
Esmaeilzadeh	Neonatal intensive care unit admission	Low	Low	Low	Low	Low	Low
Esmaeilzadeh	OGTT 0'	Low	Low	Low	Low	Low	Low
Farren	GDM	Low	Some concerns	Low	Low	Some concerns	Some concerns
Farren	OGTT 0'	Low	Some concerns	Low	Low	Some concerns	Some concerns
Farren	OGTT 60'	Low	Some concerns	Low	Low	Some concerns	Some concerns
Farren	OGTT 120'	Low	Some concerns	Low	Low	Some concerns	Some concerns
Farren	Gestational age at birth	Low	Some concerns	Low	Low	Some concerns	Some concerns
Farren	Birth weight	Low	Some concerns	Low	Low	Some concerns	Some concerns
Farren	Macrosomia	Low	Some concerns	Low	Low	Some concerns	Some concerns
Farren	C-section rate	Low	Some concerns	Low	Low	Some concerns	Some concerns
Farren	pregnancy-induced hypertensive disorders	Low	Some concerns	Low	Low	Some concerns	Some concerns
Farren	Preterm birth	Low	Some concerns	Low	Low	Some concerns	Some concerns
Farren	Shoulder dystocia	Low	Some concerns	Low	Low	Some concerns	Some concerns

Farren	Neonatal intensive care unit admission	Low	Some concerns	Low	Low	Some concerns	Some concerns
Farren	Neonatal hypoglycemia	Low	Some concerns	Low	Low	Some concerns	Some concerns
Matarelli	GDM	Some concerns	Low	Low	Low	Some concerns	Some concerns
Matarelli	Insulin therapy	Some concerns	Low	Low	Low	Low	Some concerns
Matarelli	Neonatal hypoglycemia	Some concerns	Low	Low	Low	Low	Some concerns
Matarelli	OGTT 0'	Some concerns	Low	Low	Low	Low	Some concerns
Matarelli	OGTT 60'	Some concerns	Low	Low	Low	Low	Some concerns
Matarelli	OGTT 120'	Some concerns	Low	Low	Low	Low	Some concerns
Matarelli	Gestational age at birth	Some concerns	Low	Low	Low	Low	Some concerns
Matarelli	Birth weight	Some concerns	Low	Low	Low	Low	Some concerns
Santamaria	GDM	Some concerns	Some concerns	Some concerns	Low	Some concerns	Some concerns
Santamaria	OGTT 0'	Some concerns	Some concerns	Some concerns	Low	Some concerns	Some concerns
Santamaria	OGTT 60'	Some concerns	Some concerns	Some concerns	Low	Some concerns	Some concerns
Santamaria	OGTT 120'	Some concerns	Some concerns	Some concerns	Low	Some concerns	Some concerns
Santamaria	Gestational age at birth	Some concerns	Some concerns	Some concerns	Low	Some concerns	Some concerns
Santamaria	Birth weight	Some concerns	Some concerns	Some concerns	Low	Some concerns	Some concerns
Santamaria	C-section rate	Some concerns	Some concerns	Some concerns	Low	Some concerns	Some concerns
Santamaria	Macrosomia	Some concerns	Some concerns	Some concerns	Low	Some concerns	Some concerns
Santamaria	Preterm birth	Some concerns	Some concerns	Some concerns	Low	Some concerns	Some concerns
Santamaria	pregnancy-induced hypertensive disorders	Some concerns	Some concerns	Some concerns	Low	Some concerns	Some concerns
Santamaria	Neonatal intensive care unit admission	Some concerns	Some concerns	Some concerns	Low	Some concerns	Some concerns

Santamaria	Shoulder dystocia	Some concerns	Some concerns	Some concerns	Low	Some concerns	Some concerns
Santamaria	Insulin therapy	Some concerns	Some concerns	Some concerns	Low	Some concerns	Some concerns
Santamaria	Neonatal hypoglycemia	Some concerns	Some concerns	Some concerns	Low	Some concerns	Some concerns
Vitale	OGTT 0'	Low	Some concerns	Low	Low	Some concerns	Some concerns
Vitale	OGTT 60'	Low	Some concerns	Low	Low	Some concerns	Some concerns
Vitale	OGTT 120'	Low	Some concerns	Low	Low	Some concerns	Some concerns
Vitale	GDM	Low	Some concerns	Low	Low	Some concerns	Some concerns
Vitale	pregnancy-induced hypertensive disorders	Low	Some concerns	Low	Low	Some concerns	Some concerns
Vitale	Insulin need	Low	Some concerns	Low	Low	Some concerns	Some concerns

Table S4. GRADE: The quality of evidence in the inositol treated groups compared to placebo

Certainty assessment							№ of patients		Effect		Certainty	Importance
№ of studies	Study design	Risk of bias	Inconsistency	Indirectness	Imprecision	Other considerations	inositol	placebo	Relative (95% CI)	Absolute (95% CI)		
**GDM**												
8	randomised trials	serious	serious	not serious	not serious	strong association	100/698 (14.3%)	195/659 (29.6%)	**RR 0.42** (0.26 to 0.67)	**172 fewer per 1 000** (from 219 fewer to 98 fewer)	⨁⨁⨁◯ Moderate	
**Glucose 0' (assessed with: mmol/l or mg/dl)**												

Certainty assessment							№ of patients		Effect		Certainty	Importance
№ of studies	Study design	Risk of bias	Inconsistency	Indirectness	Imprecision	Other considerations	inositol	placebo	Relative (95% CI)	Absolute (95% CI)		
8	randomised trials	serious	serious	not serious	not serious	none	698	659	-	MD **0.17 mmol/l lower** (0.26 lower to 0.09 lower)	⊕⊕◯◯ Low	
**Glucose 60' (assessed with: mmol/l or mg/dl)**												
7	randomised trials	serious	serious	not serious	not serious	none	671	630	-	MD **0.44 mmol/l lower** (0.74 lower to 0.14 lower)	⊕⊕◯◯ Low	
**Glucose 120' (assessed with: mmol/l or mg/dl)**												

Certainty assessment							№ of patients		Effect		Certainty	Importance
**№ of studies**	**Study design**	**Risk of bias**	**Inconsistency**	**Indirectness**	**Imprecision**	**Other considerations**	**inositol**	**placebo**	**Relative (95% CI)**	**Absolute (95% CI)**		
7	randomised trials	serious	serious	not serious	not serious	none	671	630	-	MD **0.37 mmol/l lower** (0.69 lower to 0.06 lower)	⊕⊕◯◯ Low	

Insulin therapy

6	randomised trials	serious	not serious	not serious	serious	none	24/469 (5.1%)	48/438 (11%)	**RR 0.45** (0.28 to 0.73)	**60 fewer per 1 000** (from 79 fewer to 30 fewer)	⊕⊕◯◯ Low	

Birthweight (assessed with: g)

Certainty assessment							№ of patients		Effect		Certainty	Importance
№ of studies	Study design	Risk of bias	Inconsistency	Indirectness	Imprecision	Other considerations	inositol	placebo	Relative (95% CI)	Absolute (95% CI)		
6	randomised trials	serious	serious	not serious	very serious	none	561	517	-	MD **4.83 lower** (96.88 lower to 87.21 higher)	⊕◯◯◯ Very low	
**Gestational age at birth**												
6	randomised trials	serious	very serious	not serious	not serious	none	561	517	-	MD **0.52 higher** (0.03 lower to 1.08 higher)	⊕◯◯◯ Very low	
**Macrosomia**												

Certainty assessment							№ of patients		Effect		Certainty	Importance
№ of studies	Study design	Risk of bias	Inconsistency	Indirectness	Imprecision	Other considerations	inositol	placebo	Relative (95% CI)	Absolute (95% CI)		
5	randomised trials	serious	serious	not serious	very serious	none	22/435 (5.1%)	28/450 (6.2%)	**RR 0.74** (0.28 to 1.99)	**16 fewer per 1 000** (from 45 fewer to 62 more)	⨁◯◯◯ Very low	
**C-section rate**												
6	randomised trials	serious	not serious	not serious	very serious	none	219/540 (40.6%)	227/502 (45.2%)	**RR 0.90** (0.78 to 1.03)	**45 fewer per 1 000** (from 99 fewer to 14 more)	⨁◯◯◯ Very low	

Certainty assessment							№ of patients		Effect		Certainty	Importance
**№ of studies**	**Study design**	**Risk of bias**	**Inconsistency**	**Indirectness**	**Imprecision**	**Other considerations**	**inositol**	**placebo**	**Relative (95% CI)**	**Absolute (95% CI)**		
**Neonatal hypoglycaemia**												
5	randomised trials	serious	serious	not serious	serious	none	17/449 (3.8%)	24/413 (5.8%)	**RR 0.53** (0.10 to 2.71)	**27 fewer per 1 000** (from 52 fewer to 99 more)	⨁◯◯◯ Very low	
**NICU admission**												

Certainty assessment							№ of patients		Effect		Certainty	Importance
**№ of studies**	**Study design**	**Risk of bias**	**Inconsistency**	**Indirectness**	**Imprecision**	**Other considerations**	**inositol**	**placebo**	**Relative (95% CI)**	**Absolute (95% CI)**		
4	randomised trials	serious	not serious	not serious	serious	none	8/414 (1.9%)	14/375 (3.7%)	**RR 0.60** (0.24 to 1.47)	**15 fewer per 1 000** (from 28 fewer to 18 more)	⊕⊕◯◯ Low	
**Preterm birth**												
6	randomised trials	serious	not serious	not serious	not serious	strong association	15/540 (2.8%)	36/502 (7.2%)	**RR 0.41** (0.22 to 0.75)	**42 fewer per 1 000** (from 56 fewer to 18 fewer)	⊕⊕⊕⊕ High	

Certainty assessment							№ of patients		Effect		Certainty	Importance
№ of studies	Study design	Risk of bias	Inconsistency	Indirectness	Imprecision	Other considerations	inositol	placebo	Relative (95% CI)	Absolute (95% CI)		
**Shoulder dystocia**												
3	randomised trials	serious	not serious	not serious	very serious	none	2/291 (0.7%)	4/304 (1.3%)	**RR 0.59** (0.12 to 2.82)	**5 fewer per 1 000** (from 12 fewer to 24 more)	⨁◯◯◯ Very low	
**Pregnancy-induced hypertensive disorders**												
7	randomised trials	serious	not serious	serious	serious	strong association	17/650 (2.6%)	46/615 (7.5%)	**RR 0.39** (0.22 to 0.69)	**46 fewer per 1 000** (from 58 fewer to 23 fewer)	⨁⨁◯◯ Low	

CI: confidence interval; **MD:** mean difference; **RR:** risk ratio

REFERENCES

1. Celentano C, Matarrelli B, Pavone G, Vitacolonna E, Mattei PA, Berghella V, et al. The influence of different inositol stereoisomers supplementation in pregnancy on maternal gestational diabetes mellitus and fetal outcomes in high-risk patients: a randomized controlled trial. J Matern Fetal Neonatal Med. 2020;33(5):743-51.
2. D'Anna R, Scilipoti A, Giordano D, Caruso C, Cannata ML, Interdonato ML, et al. myo-Inositol supplementation and onset of gestational diabetes mellitus in pregnant women with a family history of type 2 diabetes: a prospective, randomized, placebo-controlled study. Diabetes Care. 2013;36(4):854-7.
3. D'Anna R, Di Benedetto A, Scilipoti A, Santamaria A, Interdonato ML, Petrella E, et al. Myo-inositol Supplementation for Prevention of Gestational Diabetes in Obese Pregnant Women: A Randomized Controlled Trial. Obstet Gynecol. 2015;126(2):310-5.
4. Esmaeilzadeh S, Ghadimi R, Mashayekh-Amiri S, Delavar MA, Basirat Z. The effect of myo-inositol supplementation on the prevention of gestational diabetes in overweight pregnant women: a randomized, double-blind, controlled trial. Minerva Obstet Gynecol. 2022.
5. Farren M, Daly N, McKeating A, Kinsley B, Turner MJ, Daly S. The Prevention of Gestational Diabetes Mellitus With Antenatal Oral Inositol Supplementation: A Randomized Controlled Trial. Diabetes Care. 2017;40(6):759-63.
6. Matarrelli B, Vitacolonna E, D'Angelo M, Pavone G, Mattei PA, Liberati M, et al. Effect of dietary myo-inositol supplementation in pregnancy on the incidence of maternal gestational diabetes mellitus and fetal outcomes: a randomized controlled trial. J Matern Fetal Neonatal Med. 2013;26(10):967-72.
7. Santamaria A, Di Benedetto A, Petrella E, Pintaudi B, Corrado F, D'Anna R, et al. Myo-inositol may prevent gestational diabetes onset in overweight women: a randomized, controlled trial. J Matern Fetal Neonatal Med. 2016;29(19):3234-7.
8. Vitale SG, Corrado F, Caruso S, Di Benedetto A, Giunta L, Cianci A, et al. Myo-inositol supplementation to prevent gestational diabetes in overweight non-obese women: bioelectrical impedance analysis, metabolic aspects, obstetric and neonatal outcomes - a randomized and open-label, placebo-controlled clinical trial. Int J Food Sci Nutr. 2021;72(5):670-9.
